# High quality 3C *de novo* assembly and annotation of a multidrug resistant ST-111 *Pseudomonas aeruginosa* genome: Benchmark of hybrid and non-hybrid assemblers

**DOI:** 10.1038/s41598-020-58319-6

**Published:** 2020-01-29

**Authors:** José Arturo Molina-Mora, Rebeca Campos-Sánchez, César Rodríguez, Leming Shi, Fernando García

**Affiliations:** 1 Centro de Investigación en Enfermedades Tropicales, Facultad de Microbiología, Universidad de Costa Rica, San José, Costa Rica; 2 Centro de Investigación en Biología Celular y Molecular, Facultad de Microbiología, Universidad de Costa Rica, San José, Costa Rica; 3 Human Phenome Institute of Fudan University, Shanghai, China

**Keywords:** Computational biology and bioinformatics, Genome informatics, Genome assembly algorithms

## Abstract

Genotyping methods and genome sequencing are indispensable to reveal genomic structure of bacterial species displaying high level of genome plasticity. However, reconstruction of genome or assembly is not straightforward due to data complexity, including repeats, mobile and accessory genetic elements of bacterial genomes. Moreover, since the solution to this problem is strongly influenced by sequencing technology, bioinformatics pipelines, and selection criteria to assess assemblers, there is no systematic way to select *a priori* the optimal assembler and parameter settings. To assembly the genome of *Pseudomonas aeruginosa* strain AG1 (PaeAG1), short reads (Illumina) and long reads (Oxford Nanopore) sequencing data were used in 13 different non-hybrid and hybrid approaches. PaeAG1 is a multiresistant high-risk sequence type 111 (ST-111) clone that was isolated from a Costa Rican hospital and it was the first report of an isolate of *P. aeruginosa* carrying both blaVIM-2 and blaIMP-18 genes encoding for metallo-β-lactamases (MBL) enzymes. To assess the assemblies, multiple metrics regard to contiguity, correctness and completeness (3C criterion, as we define here) were used for benchmarking the 13 approaches and select a definitive assembly. In addition, annotation was done to identify genes (coding and RNA regions) and to describe the genomic content of PaeAG1. Whereas long reads and hybrid approaches showed better performances in terms of contiguity, higher correctness and completeness metrics were obtained for short read only and hybrid approaches. A manually curated and polished hybrid assembly gave rise to a single circular sequence with 100% of core genes and known regions identified, >98% of reads mapped back, no gaps, and uniform coverage. The strategy followed to obtain this high-quality 3C assembly is detailed in the manuscript and we provide readers with an all-in-one script to replicate our results or to apply it to other troublesome cases. The final 3C assembly revealed that the PaeAG1 genome has 7,190,208 bp, a 65.7% GC content and 6,709 genes (6,620 coding sequences), many of which are included in multiple mobile genomic elements, such as 57 genomic islands, six prophages, and two complete integrons with blaVIM-2 and blaIMP-18 MBL genes. Up to 250 and 60 of the predicted genes are anticipated to play a role in virulence (adherence, quorum sensing and secretion) or antibiotic resistance (β-lactamases, efflux pumps, etc). Altogether, the assembly and annotation of the PaeAG1 genome provide new perspectives to continue studying the genomic diversity and gene content of this important human pathogen.

## Introduction

Genotyping methods and genome sequencing are indispensable to reveal genomic structure and evolution of bacterial clones with high resolution^1^. In this sense, production of large amounts of short sequencing data from genomes (reads) has been facilitated by continuous advances in Next Generation Sequencing (NGS) technologies. This includes short read sequencing technologies (a few hundred bp read length) such as Illumina and long read sequencing technologies (several hundred kb read length) such as Pacific Biosciences (PacBio) single-molecule real-time (SMRT) and Oxford Nanopore Technology (ONT)^2^.

Using sequencing data, it is expectable that full-length chromosomes could be produced when the genome is fully sequenced and assembled^3^. However, reconstruction of genome or assembly is not straightforward due data complexity. This is a challenging problem that requires time and expertise^4^. If a reference genome is available, an assembly can be made by comparison or direct mapping, otherwise, a *de novo* assembly, in which only the information obtained from reads is used to reconstruct the genome, without prior knowledge of its organization^5^. In *de novo* assembly, sequences (reads) are grouped into contigs using graph based algorithms such as Overlap-Layout-Consensus, *de Bruijn* and greedy approaches^5,6^. Then contigs are assembled into scaffolds (supercontigs or metacontigs). Alternatively, some *de novo* assemblers use reference genomes to solve specific inconsistencies or for scaffolding^5^.

Reconstruction can be favored by some previous information, such as expected genome size, GC content and repetitive region content, as they help choose the best strategy to follow. Even though many algorithms to assemble genome by *de novo* approaches are available, performance is completely dependent on data (short or long reads, instruments, technology), genomic complexity (repeats, number of chromosomes or plasmids) and complementary algorithms (pre-processing, databases, annotations, etc)^7^. Therefore, for a specific genome and dataset, selection of the optimal assembly strategy to use is not a trivial task because there is no systematic way to determine which assembler and what parameter settings must be selected^8^.

Since a key first requirement in the study of genomes is accuracy^9^, short reads technologies are preferred because they produce high fidelity reads^10^. Also, the low cost and high accuracy of Illumina sequencing makes it well suited to high-throughput bacterial genomics^10^. However, genomes present complex repeat structures difficult to solve by different assemblers. As reported, if the repeats are longer than the reads, genomic regions sharing perfect repeats can be indistinguishable^6^. With this, resolving a full genome is a challenging issue for short reads approaches. Consequently, most available bacterial genomes are incomplete^11^, highly fragmented, and of low quality^3^.

Long reads, by contrast, can exceed the length of repeats in a typical bacterial genome, facilitating genome assembly^10^. Long reads technology offers an important advantage for complex genomes with high level of repetitive elements or genome duplication^7^. Thus, use of long reads data has shown improvements in the context of *de novo* genome assemblies, rising contiguity, solving fragmented regions, and closing gaps^12^. However, these third generation sequencing methods deal with relatively high sequencing error^8^, which has been estimated up to 15% of random but also systematic errors^10,12^. In addition, long reads sequencing has a higher cost per base than that with Illumina platforms^11^.

Combination of reads of different length and from different sequencing platforms in so-called hybrid approaches often counterbalances the drawbacks of each method^4^. The growing interest in hybrid assemblies is justified by the popularity, cost and accuracy of short reads sequencing, plus the resolution capacity of repetitive regions and genomic structures of long reads^10^. In some cases, a hybrid approach is sufficient to produce a single and closed sequence of the microbial genome^13^. However, to accurately assemble a genome, neither the optimum combination and coverage of long and short reads, nor the minimum required length of long-reads are known a priori^9^. Due to this, hybrid and non-hybrid assembly must be individually evaluated with regard to select the best assembly conditions, and different metrics and tools are available for this purpose. However, no single or completely useful strategy is considered as universal and sufficient to benchmark assemblies^3,14^.

Benchmark of assemblies can be achieved using metrics related to contigs and scaffolds (contiguity), ability to complete the whole structure of the genome (completeness), and the accuracy of the assembly (correctness). Although most of studies of assemblies exploit these parameters to evaluate the performance of assemblers^3,8,10,15–17^, here we define the general assessment by “3C criterion” as all metrics required to evaluate and benchmark genome assemblies using contiguity, completeness and correctness metrics, as detailed:

- Contiguity: It evaluates the assembly in terms of number and size of contigs and scaffolds^6^, the pieces found in an assembly. Metrics includes statistics related to maximum length, average length, combined total length, and contig N50 (length-weighted median of ordered contigs or scaffolds)^2^. However, contiguity metrics thereof need to be interpreted with caution due they do not contain information on assembly accuracy and completeness^4^.
- Correctness: it refers to how well those pieces accurately represent the genome sequenced^16^ and, in general is acceptable that it is essential to prioritize correctness rather than contiguity^12^. However, correctness is difficult to evaluate if a preliminary reference genome is not available, which is a particular problem for *de novo* assembly^6^. Mapping and comparison to reference or draft genome (or a consensus sequence) can be used to detect misassemblies, including mismatches, indels, and misjoins^8^.
- Completeness: it assesses how much of the genome is represented by the pieces of the assembly^16^. This implies the evaluation of ability to assembly not only all the genes, but also to solve all complicated regions, including repetitive sequences and, if it is expected, circularization of genome. The most important metric for this case is the “completeness score”, calculated by the examination of single-copy orthologs conserved genes^18^. In addition, information of known sequences, unexpected variations in coverage, and remapping of reads allows to analyze the consistency of the genome and identification of potentially poorly assembled regions^5,19^.

Thus, to develop a strategy to assembly a bacterial genome using the non-hybrid and hybrid approaches as well as the 3C criterion, we used a ST-111 strain of *Pseudomonas aeruginosa*. *P. aeruginosa* is Gram-negative bacterium and a well-known opportunistic pathogen^20^. It is responsible for acute and chronic nosocomial and community infections in immune-compromised patients^21^. However, the treatment of *P. aeruginosa* infections is challenging due many intrinsic and acquired mechanisms of resistance^22^, including the production of to β-lactamases antibiotic modifying enzymes and target alteration.

Multi-resistance in *P. aeruginosa* is becoming more and more serious, not only due resistance to classical β-lactams, aminoglycosides and fluoroquinolones, but also to resistance last resort treatments including carbapenems (β-lactams) and colistin, which causes great difficulties in clinical treatment^23,24^ and resistant to these antibiotics emerge as a final level of fight of bacteria which compromises infections treatments^24^. Many bacterial clones with carbapenemase-producing features are recognized as high-risk clones^25^. A high-risk clone is a multidrug-resistant clone with highly efficient transmission and/or maintenance among humans or animals^26^, playing a major role in the spread of resistance in the hospital and other environments^27^ and a flexible ability to accumulate and switch resistance^28^. However, the term high-risk is not necessarily associated with severity^26^. A limited number of *Pseudomonas aeruginosa* genotypes (mainly ST-111, ST-175, and ST-235) are recognized as high-risk clones, and they are responsible for epidemics of nosocomial infections by multidrug-resistant or extensively drug-resistant strains worldwide^29^.

In Costa Rica, isolation of carbapenem resistant *P. aeruginosa* strains is relatively common in some major hospitals as we reported before^22^, most of them carrying one blaVIM and one blaIMP allele carbapenemases and up to 63.1% of prevalence^22^, much higher than the frequencies observed in other countries^30^.

The Costa Rican multi-resistant strain *P. aeruginosa* AG1 (PaeAG1) was isolated from a sputum sample of a patient with pneumonia from the Intensive Care Unit of the San Juan de Dios Hospital (San José, Costa Rica) in 2010. PaeAG1 has a resistance phenotype to β-lactam (including carbapenems), aminoglycosides and fluoroquinolones, showing susceptibility only to colistin. In addition, PaeAG1 was identified as the first report worldwide of a strain carrying both blaIMP-18 (or IMP-18) and blaVIM-2 (VIM-2) genes, coding for metallo-β-lactamases (MBL) with carbapenemase activity^22^.

PaeAG1 is a high-risk clone with a genotyping profile ST-111, which includes strains with a phenotype extremely resistant to antibiotics, responsible for various types of infections in hospitals and rapid spread between the individuals^29,31^. Sanger sequencing confirmed that the blaVIM-2 and blaIMP-18 genes of strain AG1 (Accessions KC907377 and KC907378) are encoded in class 1 integrons, likely in two different structures^22^. In addition, preliminary experimental assays suggested no existence of plasmids^22^.

We were interested in assembling and annotating the genome of the clinical isolate PaeAG1 due to its importance as a high-risk clone with multi-resistance to antibiotics and to identify molecular determinants related to the ability to conquer nosocomial environments, virulence and other phenotypes. Thus, the aims of our study were: (i) to assemble the PaeAG1 genome using short and long reads data by hybrid and non-hybrid multiple approaches, (ii) to benchmark assemblers and select the best genome assembly approach using the 3C criterion, and (iii) to annotate the PaeAG1 genome to characterize and identify general gene content and genomic determinants associated with its multidrug-resistance and virulence phenotypes.

## Methods

**Figure 1:**
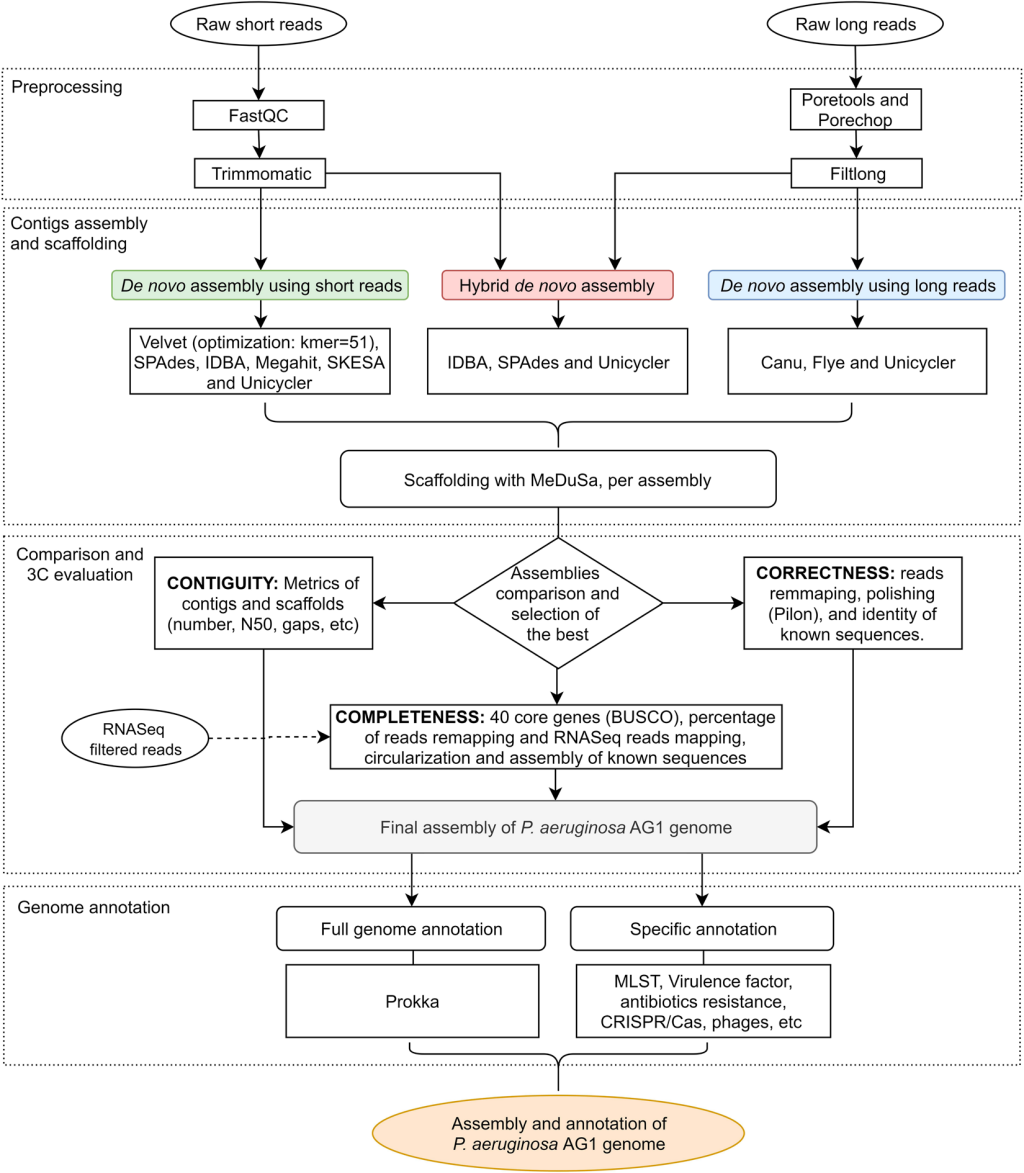
General bioinformatic pipeline to assemble, compare and annotate the *Pseudomonas aeruginosa* AG1 genome using short and long reads as well as hybrid approaches.

The general pipeline followed to assembly the PaeAG1 genome by hybrid and non-hybrid approaches is shown in Fig. 1. Complete details of settings of implemented algorithms are shown in supplementary material “Scripts for bioinformatics analysis”.

### Bacterial isolate

The Costa Rican PaeAG1 strain was isolated in 2010 from a sputum sample of a patient with pneumonia from the Intensive Care Unit of the San Juan de Dios Hospital (San José, Costa Rica). This isolate has phenotypic resistance (AST-GN cards, bioMeriux Vitek) to β-lactams, aminoglycosides and fluoroquinolones, shows susceptibility only to colistin and expresses metallo-β-lactamase activity (E-test MBL strips, AB Biodisk), as reported^22^.

### Bacterial growth and DNA isolation

PaeAG1 cells were grown overnight in Luria-Bertani broth (LB) medium at 37 °C with shaking. Then, cells were collected by centrifugation and genomic DNA was isolated with the QIAGEN DNeasy Kit (QIAGEN, UK) following the manufacturer’s instructions.

The yield of genomic DNA obtained was determined using a Nanodrop (Nanodrop 2000, Thermo Scientific, UK) and by Qubit Fluorometric Quantitation (Qubit 3.0 Fluorometer, Thermo Scientific). DNA integrity was verified by electrophoresis using 0.7% agarose gels.

### Whole genome sequencing using short reads

Genomic DNA was sequenced using Illumina technology (Illumina Inc.) at Macrogen, Korea. The sequencing library was prepared using TruSeq DNA Sample Prep kit with the standard Illumina DNA shotgun library preparation protocol. DNA fragmentation was achieved by ultrasonication, and then adapter ligation and PCR enrichment were done. Paired end reads of 101 bp were generated using a HiSeq. 2000 sequencing instrument. Sequence files were evaluated using FastQC v0.11.7^32^ before and after trimming. Reads were trimmed (including adapters removal) using Trimmomatic v0.38^33^ to discard sequences with per base sequence quality score <30. After selection, 7.4 Gb of sequences were kept, with a 14 million of pairs of reads and mean coverage >400X according to expected genome size (approx. 7 Mb).

### Whole genome sequencing using long reads

Long reads from genomic DNA was sequenced using Oxford Nanopore technology by NextOmics, Wuhan-China. Sequencing libraries were prepared according to the ONT 1D ligation library protocolSQK-LSK109. FLO-MIN-106 flowcell and the standard 48-hour run script with active channel selection enabled were used to sequence reads in a GridION instrument. Poretools v0.6.0^34^ was used to extract and evaluate reads by quality before and after trimming. Adapters were removed using Porechop v0.2.3 (github.com/rrwick/Porechop) and trimming was done using Filtlong v0.2.0 (github.com/rrwick/Filtlong). Reads with mean quality weight <10 and/or shorter than 1 kb were discarded. The final dataset consisted of 4.5 Gb of sequence, with 259,491 reads in total, a read mean length of 17,343 bp, a longest read of 201,659 bp, and a final mean coverage >560X.

### Short reads genome assembly

Six *de Bruijn* graph based assemblers were used with default parameters and without reference guided option, if applicable. The classical assemblers included in the study were Velvet v1.2.10^35^, SPAdes v3.13.0^36^, IDBA v1.1.3^37^, and Megahit v1.1.3^38^. Two newer assemblers were also included: SKESA v2.3.0^39^ and Unicycler v0.4.7^11^. To estimate the best k-mer length for genome *de novo* assembly for Velvet, KmerGenie 1.7051 was implemented^40^. Other algorithms selected best k-mer length values automatically, if needed. Assembly sequences were kept at contig level with minimum size of 1,000 bp.

### Long reads genome assembly

Three graph-based long read assemblers were used: Canu 1.8^41^, Flye 2.3.7^42^ and Unicycler v0.4.7^11^. Default parameters and no reference genome nor alternative sequencing data were considered. Only contigs with size higher than 1,000 bp were kept.

### Hybrid genome assembly

Three graph-based hybrid approaches were applied. Default parameters without reference sequence were used to run IDBA-hyb v1.1.1 (https://github.com/loneknightpy/idba), Unicycler v0.4.7^11^ and SPAdes v3.13.0^43^. Only contigs with size higher than 1,000 bp were kept.

### Scaffolding

Prior the final version of the genome assembly of PaeAG1, BLASTn (blast.ncbi.nlm.nih.gov/Blast.cgi) was used to search closest genome according to contig sequences. All assemblies at contig level were assembled into scaffolds using the closest genome as reference sequences (*P. aeruginosa* strain RIVM-EMC2982, more details in Results) using MeDuSa v1.6^44^. When final version was achieved, scaffolding and benchmarking was done using the definitive version of the PaeAG1 genome with same scaffolder.

### 3C Benchmark of approaches and selection of best assembly

Benchmark of all assemblers were done according to 3C criterion, as follow:

#### Contiguity

Genome assembly statistics about quality and contiguity were assessed using QUAST 5.0.1^14^ at both contig and scaffold levels. Assembler outputs were compared with regards to total assembly length (expected: around 7 Mb), number of contigs/scaffolds (one sequence expected), N50 (expected: as large as possible, close to genome size), NG50 (as large as possible), and others.

#### Completeness

Four strategies were implemented to assess completeness. First, single copy ortholog gene sets were searched (expected: 100%) in the assemblies using the BUSCO tool^45^ within the gVolante plataform (https://gvolante.riken.jp)^18^ and comparing gene content against 40 genes of the bacteria database (available at https://busco.ezlab.org/v1/). We also checked the ability of the assemblers to reproduce the complete sequences of the two class I integrons of PaeAG1 previously obtained by Sanger sequencing (KC907377 and KC907378). The third analysis used Circlator^19^ to assess the replicon circularization achieved by assemblers that gave rise to single sequences (expected: a circular sequence). A last approach calculated the percentage of genomic and transcriptomic reads mapping to each genome reconstruction (expected: >95% mapping). To this end, short and long reads were remapped to the assemblies using BWA 0.7.17^46^. In addition, 12 reads files from a RNASeq experiment (triplicates of same strain under four experimental conditions with or without ciprofloxacin) were mapped to the assemblies using HISAT2 v2.1.0^47^. Qualimap v2.2.2^48^ was used to calculate coverage and percentage of mapped reads, and comparison was done in a single report using MultiQC v1.7^49^.

#### Correctness

Two strategies were used to evaluate correctness. The first one was to estimate error rates, check for uniform coverage, and detect false variants of short reads that mapped to the polished genome (see below, expected: 0% errors). This was done using Qualimap results. The second strategy was to calculate the percentage of identity of local alignments between known Sanger sequences (integrons, expected: 100% identity) of PaeAG1 and the final assembly (BLASTn).

All above criteria were considered to select the best assembly. This draft genome was polished and curated (next section) and the new version was included as extra 13^th^ assembly.

We used all quantitative data to run a Principal Components Analysis (PCA), which was implemented in R software v3.5.1 (www.r-project.org/) using the Carret package (caret.r-forge.r-project.org/). This let to compare global profiles and performance given by assemblers. The final version of genome assembly was also included as an independent unit.

*De novo* assembly graphs were visualized using Bandage v0.8.1^50^. Finally, assembled sequences were visualized and compared against the final assembly using the BLAST Ring Image Generator (BRIG) tool v0.95^51^.

### Curation and polishing of the definitive genome assembly

Final adjustments of selected genome assembly were made manually based on the assembly graph, read coverage and distribution. Pilon 1.23^52^ with BWA-mapped reads were implemented to automatically polish the assemblies. After this, a final polished assembly was obtained. Remapping of short and long reads, as well as all metrics calculations and 3C criterion evaluations were done again.

### Comparative genome analysis

BLASTn of complete sequence was run again to find the closest genome, which jointly with the genome of the reference strain *P. aeruginosa* PAO1 were compared using Mauve v2.4.0^53^ to determine the level of synteny and to describe global genomic structure.

Also, in order to compare the PaeAG1 genome with other ST-111 strains, a phylogenetic analysis was done using all the available complete sequences of ST-111 *P. aeruginosa* genomes. The reference strain *P. aeruginosa* PAO1 was also included. All the records were retrieved from Pseudomonas Genomes Database (PGDB, pseudomonas.com), and Roary program v3.12.0^54^ was run with default parameters to establish relationships between strains using gene content by a pan-genome analysis. Scripts supplied with the program were used to create plots.

### Whole genome annotation

For all assemblies, gene prediction and gene annotation was achieved using Prokka v1.13.3^55^ and a custom database created with the genome of *P. aeruginosa* PAO1 and closest annotated strain to PaeAG1 as primary sources for annotation, or the default bacterial database provided with the software distribution. Also, Clusters of Orthologous Groups (COG), Gene Ontology (GO) and Kyoto Encyclopedia of Genes and Genomes (KEGG) pathway were searched using EggNOG (http://eggnogdb.embl.de/)^56^ for all coding sequences (CDS).

### Specific genome annotation

Specific annotation and searching for specific genomic determinants was only done for the definitive final assembly. Default parameters were used in all cases. *In silico* serotyping was done using Past v1.0 (https://cge.cbs.dtu.dk/services/PAst-1.0/) and multilocus sequence typing^57^ using MLST v2.0 (https://cge.cbs.dtu.dk/services/MLST/). Antimicrobial resistance genes were detected using RGI tool v5.1.0 (Resistance Gene Identifier, https://card.mcmaster.ca/analyze/rgi) and ResFinder v3.2 (https://cge.cbs.dtu.dk/services/ResFinder/). CRISPR-Cas arrays were investigated using CRISPRCasFinder v1.1.2 (https://crisprcas.i2bc.paris-saclay.fr/CrisprCasFinder/Index). Virulome was identified using Virulence Factor DataBase (VFDB, http://www.mgc.ac.cn/VFs/).

For mobilome delimitation, genomic islands were identified using IslandViewer v4 (www.pathogenomics.sfu.ca/islandviewer/). PHASTER was used to find prophages (phaster.ca/)^58^ and integrons were searched using IntegronFinder v2.0^59^. The results of this series of searches were visualized in the genome using BRIG.

## Results

In order to assembly the genome of *P. aeruginosa* AG1, an exhaustive workflow was implemented using hybrid and non-hybrid approaches, using Illumina short reads sequencing and Oxford Nanopore long reads sequencing data. General protocol is presented in Fig. 1. After sequencing and four bioinformatic steps, a single circular sequence was achieved and it was also annotated.

### Benchmarking of hybrid and non-hybrid assemblers: a winner?

Using different approaches, the PaeAG1 genome assembly was evaluated using the 3C criterion. The final version was presented as a last case, cured and polished. The contiguity and completeness criteria were initially the most important for the selection of the draft assembly, and then, a final polishing strategy focused on ensuring correctness (see next section). A summary of the most important metrics related to these criteria is presented in Table 1. Metrics related to scaffolding were obtained using the final assembly as reference, although various attempts to create scaffolds were made with closely related genomes.

**Table 1:** Comparison of contiguity and annotation of *P. aeruginosa* AG1 genome assembly by different approaches*.

3C Criterion	Level and metrics		Short reads only approaches					Long reads only approaches			Hybrid approaches				
			Velvet	SPAdes	IDBA	Megahit	SKESA	Unicycler	Canu	Flye	Unicycler	IDBA	SPAdes	Unicycler	Final assembly
Contiguity	Contigs assembly	Contigs	*227*	89	127	125	217	113	2	**1**	5	121	16	**1**	**1**
		Total length	*7027785*	7094145	7090598	7103650	7047434	7074438	7121028	**7209472**	**7465726**	7092836	7188777	7189601	**7190208**
		GC (%)	65.79	65.73	65.74	65.73	65.77	65.77	65.66	65.59	65.64	65.74	65.68	65.71	65.71
		N50	*65258*	223421	170948	168521	68375	151417	4329427	**7209472**	7178173	141288	1593634	**7189601**	**7190208**
		L50	33	11	14	14	34	15	1	1	1	15	2	1	1
	Scaffolding	Scaffolds	1	10	10	10	2	1	1	1	1	10	10	1	1
		N50 & NG50	7039385	7078855	7079244	7091835	7056837	7080238	7121028	7209472	7465826	7082290	7171429	7189601	7190208
		Genome fraction (%)	*97.714*	98.362	98.293	98.484	98.054	98.382	99.381	99.991	**100**	98.356	99.717	**99.992**	**100**
		NA50	177145	375326	491929	478607	708585	709611	4328063	7207242	7177177	477586	3956502	7189601	7190208
		LA50	12	6	5	5	4	4	1	1	1	4	1	1	1
		N's per 100 kbp	*217.06*	52.13	77.51	75.96	151.6	81.92	**0**	**0**	1.34	74.67	5.56	**0**	**0**
Correctness		Misassemblies	*81*	22	37	33	24	19	1	**0**	4	26	2	**0**	**0**
		Unaligned mis. contigs	0	0	0	0	0	0	0	0	0	0	0	0	0
		Mismatches per 100 kbp	6.56	2.42	4.88	1.61	1.84	**0.48**	*35.94*	*28.01*	*101.21*	3.68	11.33	**0.07**	**0**
		Indels per 100 kbp	6.49	0.41	0.67	0.28	1.79	**0.34**	*324.66*	*284.54*	*186.53*	1	1.14	**0**	**0**
Completeness	40 core genes (BUSCO)	Fragmented genes	0	0	0	0	0	0	4	9	9	0	0	0	0
		Intact genes	40	40	40	40	40	40	20	13	23	40	40	40	40
		Lost genes	0	0	0	0	0	0	*16*	*18*	*8*	0	0	0	0
		Completeness score (strict, %)	100	100	100	100	100	100	*50*	*32.5*	*57.7*	100	100	100	100
	Whole genome annotation	CDS	6574	6554	6543	6565	6540	6567	11229	9565	9089	6559	6605	6621	6620
		Contigs	1	10	10	10	2	1	1	1	1	10	10	1	1
		rRNA	2	5	5	5	3	3	12	12	12	4	14	12	12
		tmRNA	1	1	1	1	1	1	0	1	0	1	1	1	1
		tRNA	70	62	69	70	61	70	72	65	75	69	76	76	76
Completeness & correctness		Mean length of CDS (bp)	938.34	957.54	956.28	954.9	950.19	953.49	*499.35*	*607.14*	*664.14*	955.11	963.51	961.89	961.86
	Integron blaVIM-2	Identity (%)	100.0	99.5	99.8	100.0	100.0	99.7	99.488	99.257	99.843	99.753	99.778	99.778	100
		Coverage	0.5	0.7	0.6	*0.4*	0.5	0.6	**1.0**	**1.0**	1.0	0.6	**0.9**	**0.9**	**1.0**
	Integron blaIMP-18	Identity (%)	100.0	100.0	100.0	100.0	100.0	100.0	99.515	98.744	99.728	100	100	100	100
		Coverage	*0.6*	0.9	0.9	0.9	0.8	0.8	**1.0**	**1.0**	1.0	0.8	**1.0**	**1.0**	**1.0**

*For some metrics, best and worst values are marked as bold or italics, respectively.

**Figure 2:**
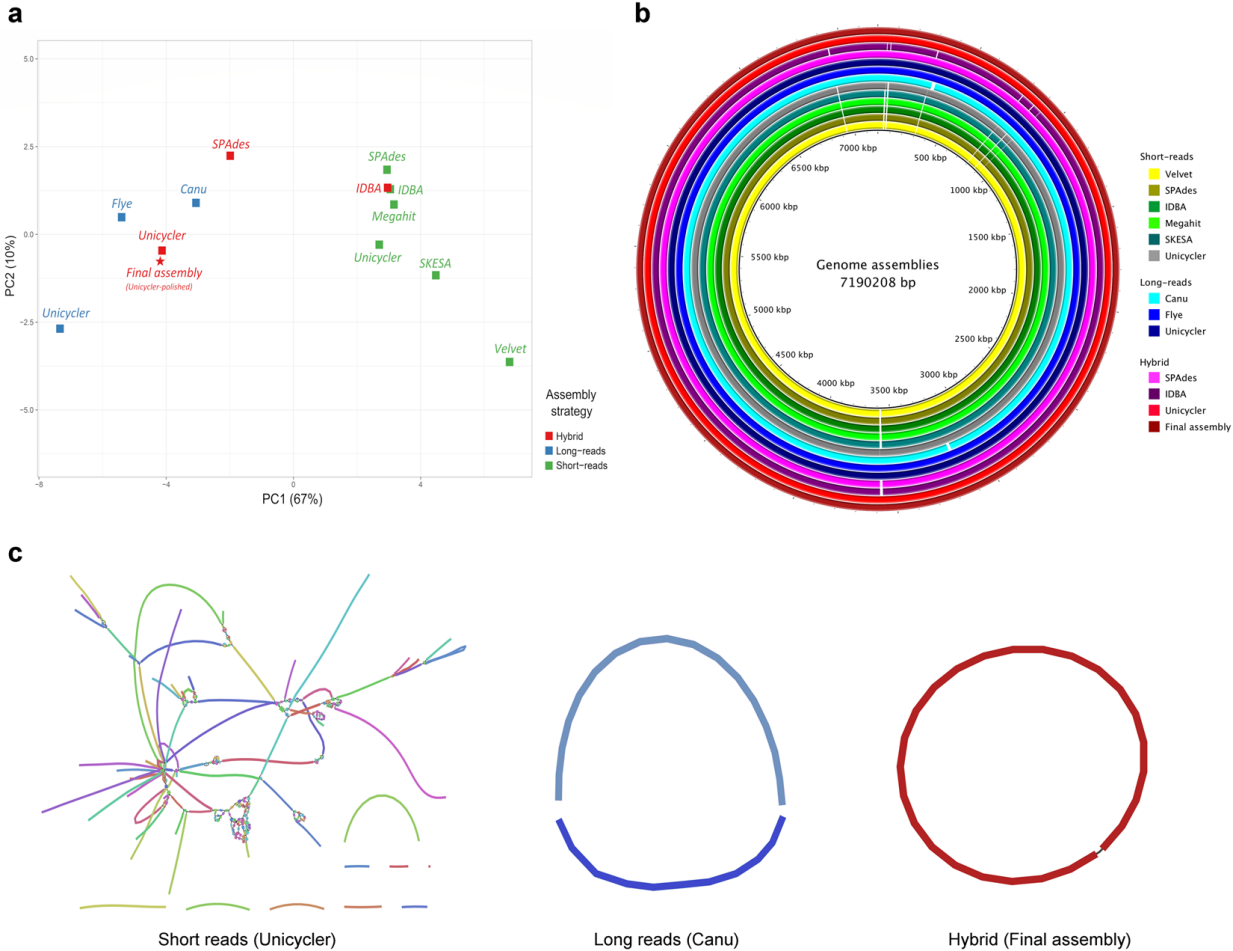
General comparison of *P. aeruginosa* AG1 genome assemblies. (**a**) Relationship between different assemblers by PCA using contiguity and annotation features. (**b**) Completeness evaluation and comparison for all different approaches using the final assembly as reference. (**c**) *De novo* assembly graph of three different approaches by short reads, long reads or hybrid assemblers. More details in Supplementary Fig. S1.

According to results of contiguity, the use of short reads only approaches shows a lower performance (89 to 227 contigs and 1–10 scaffolds) compared to other approaches that exploit long reads (1 to 5 contigs and one scaffold for all cases) or hybrid methods (1–121 contigs and 1–10 scaffolds). Performance profiles between assemblers are compared in Table 1 and Fig. 2. Short reads assemblies are similar to each other according to Table 1 and PCA (Fig. 2a). In the case of long reads approaches, hybrid or not, the performance was also similar to each other at this contiguity level. Differences depending on technology and assembly strategy are recognized according to metrics and global profiles in PCA, gaps in the assembly and graphs (Fig. 2).

Only two assemblers generated a single contig. One is a long reads only approach (Flye) and the other one is a hybrid assembler (Unicycler). The hybrid assembler IDBA obtained metrics equivalent to the mode without the use of long reads (short reads only with 127 contigs and 121 contigs for hybrid approach), and also similar to Megahit (125 contigs and other metrics). Velvet and SKESA had the higher contigs values, 227 and 217 respectively.

The anticipated total genome length was similar among the 13 assemblers (7–7.2 Mb for all cases, except for long read only Unicycler with 7.4 Mb), while the N50 value tended to be much shorter for short reads assemblies (65–171 kb) compared to long reads (4.3–7.2 Mb). However, at the scaffold level N50 values were comparable among all cases (>7.0 Mb). At this same level, all assemblies covered virtually the entire final genome, although the lower performance was obtained for short reads only approaches (>97%).

As to correctness, long reads only were linked to high rates of mismatches (28–101 per 100 kb) and indels (186–324 per 100 kb), which were not solved by posterior polishing steps (as in Unicycler). Better values were obtained for other approaches using short reads, hybrid (0–11 mismatches and 1–1.14 indels) or not (0.48–6.6 mismatches and 0.3–6.5 indels). In addition, although long reads only assemblies generated sequences of approximately the same length as the other approaches, their annotations revealed high CDS numbers (9,089–11,229, which contrast with the 6,550–6,600 for short reads and hybrid approaches). Specific analysis of sequences showed a low median CDS size (average <600 bp) from long reads only assemblers compared to other cases with short reads only or hybrid (average 955 bp, which is an expected value for PaeAG1), suggesting fragmentation of CDS in the long reads assemblies.

Evaluation of 40 core genes using BUSCO tool and completeness score showed a 100% performance for short reads only and hybrid assemblers. However, in long reads only approaches it was possible to identify 13 to 23 core genes only (32.5–57.7%).

Regarding the PaeAG1 integron sequences obtained by Sanger sequencing, with a length greater than 2,500 bp and 3,000 bp, the assemblies of short reads only had low coverage (0.4–0.9), specifically in regions with repetitions. On the other hand, models with long reads had the best performance (1.0 in all cases), and their use in the hybrid approaches improved the assembly of the aforementioned repetitive zones (0.9–1.0 for all cases, except IDBA with 0.6–0.8).

Using all information, global profiles were compared the samples using a PCA. The full table used for PCA and the components values are provided in the “Supplementary Material PCA data”. As presented in Fig. 2a, these profiles show a separation between the profiles of the short reads only (green color) and the others, creating two clusters. Also, unpolished and polished Unicycler assemblies kept close, as might be expected.

### Enhancing the winner: polishing of hybrid unicycler assembly

The assembly directly obtained from the hybrid Unicycler approach was selected as the winner for its better fulfilled the 3C criteria, and it was used for downstream analyses. However, a review of the assembly was required in evidence of: (i) missing coverage for one of the known integrons sequences (Table 1) and (ii) presence of a zone with irregular/non-uniform distribution in the remapping of long reads (Supplementary Fig. S1a -left). Due to this, a manual curation was required. Curation was carried out with the help of the known sequences of the integrons, assembly graphs, and the assemblies of long reads only (because long reads could assemble that region). A detailed explanation of the curation is provided in the “Supplementary Material Manual curation” file, including a graphical representation.

After curation with short reads, a final polishing step was carried out to guarantee completeness. Only 5 bases were modified, which is reflected in the mismatches rate (per 100 kbp) of Unicycler hybrid of 5/7,190,208*100 kb = 0.07 (Table 1). When remapping of reads was done, regular and uniform coverage was detected, even in the conflictive zone (Supplementary Fig. S1a-right). Furthermore, the known integron sequences showed complete identity and coverage (Table 1, last column).

With this improved version of the assembly, in addition to the PCA comparison, an alignment of all assemblies was done against the final assembly to highlight the problematic regions to assemble. As shown in Fig. 2b some gaps were evident in all assemblies that were derived from short reads only and these gaps were not always compensated through the use of hybrid approaches. However, for most assemblers, the use of long reads only or hybrid improved those regions. Benchmark of all assemblers in a specific conflictive region is presented in Supplementary Fig. S1b. The assembly graphs of three cases are presented in Fig. 2c, showing the variable ability of assemblers to solve the *de novo* assembly problem.

### 3C assessment of PaeAG1 final genome assembly

To assess the final assembly of PaeAG1 genome, 3C criterion was re-evaluated:

#### Contiguity

The final assembly was built with hybrid Unicycler, with curation and polishing steps, but without the need for a reference genome. Full contiguity was achieved. A single and circular sequence was obtained.

#### Completeness

With all the elements evaluated, maximum completeness is considered. This includes circularization of sequence, 100% identity and coverage of known sequences of the integrons and 100% completeness scores in 40 expected genes (single copy orthologs set). Regarding the remapping of genomic reads, 99.85% of the short reads were mapped with an average coverage of 403X (See coverage graph in Supplementary Fig. S1c left). About long reads, 97.81% were mapped to the genome with an average coverage of 560X (Supplementary Fig. S1c right). Additional data from the same strain PaeAG1 using RNASeq technology achieved a mapping of 98.6% of read sequences.

#### Correctness

The polishing rounds that Unicycler includes and the additional polishing after curation using short reads guarantee the maximum accuracy of the genome assembly.

Thus, circular assembled genome was built according to 3C criterion: high contiguity, completeness and correctness was achieved.

### Annotation of PaeAG1 genome

**Figure 3:**
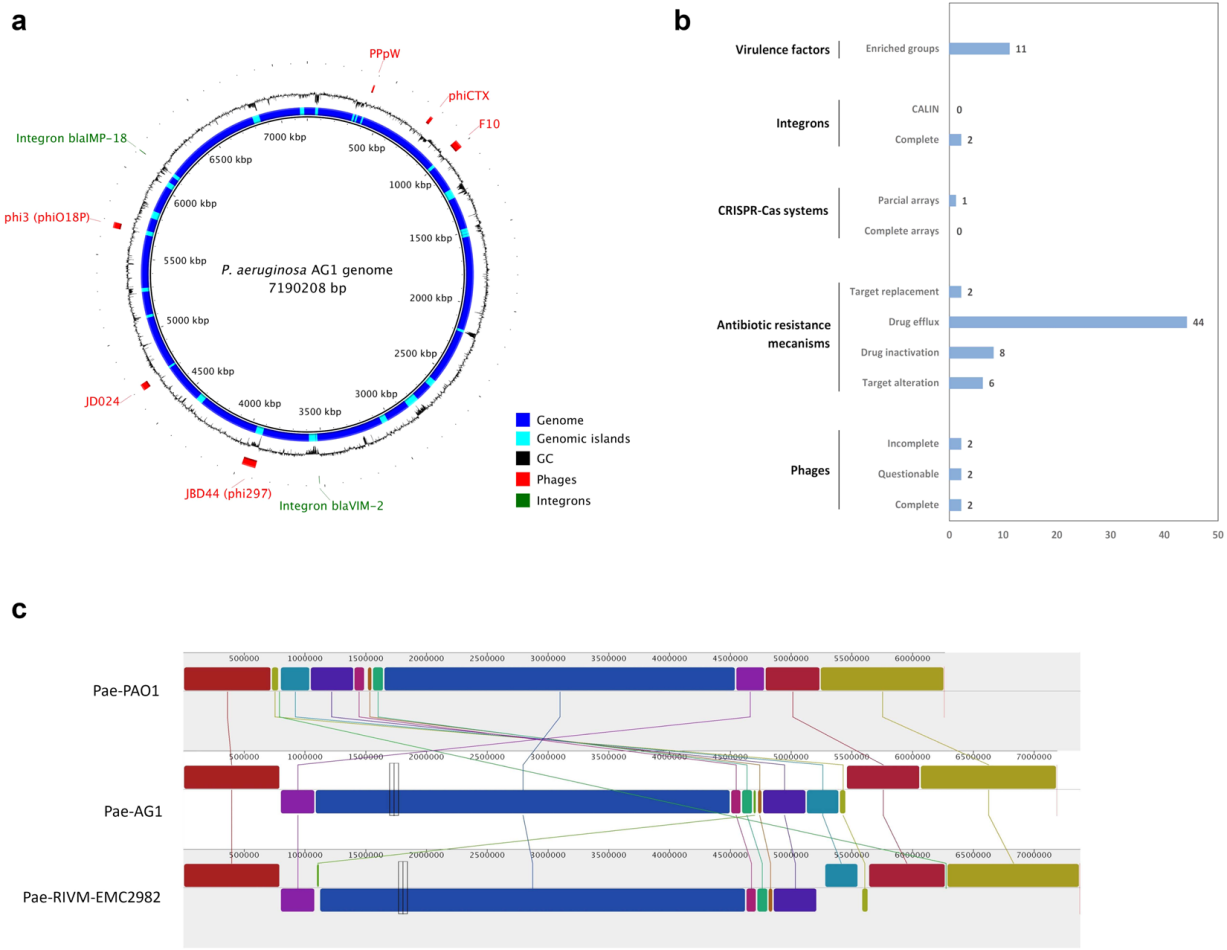
Annotation of *P. aeruginosa* AG1 genome. (**a**) Circularized genome showing phages and integrons locations. (**b**) Specific annotation of different genomic determinants including number of elements. (**c**) Genome synteny comparison among three strains of *P. aeruginosa*: PAO1 (general reference), AG1 (our assembly) and RIVM-EMC2982 (closest one to PaeAG1 according to BLAST analysis).

The PaeAG1 genome is composed of a single and circular sequence of 7,190,208 bp, with 65.71% GC content (Fig. 3a). A total of 6,620 CDS, 12 rRNA, 76 tRNA and 1 tmRNA (6,709 genes in total) were determined (Table 1). In addition, 2,197 genes were associated with Gene ontology terms, 5,537 related to defined COGs, and 3,060 to KEGG when orthologous groups and functional annotation were analyzed.

As shown in Fig. 3b, specific annotation of different genomic determinants was done, including antibiotic resistance genes, mobilome, virulence factors and others. Regarding antibiotic resistance gene profiling, genetic determinants of resistance to β-lactams, aminoglycosides, and fluoroquinolones, fosfomycin, phenicol and sulphonamide were found. By mechanism, 60 resistance associated genes were identified, including 44 efflux pumps and 8 associated with drug inactivation, including blaVIM-2 and blaIMP-18 gene alleles. Also, six determinant of target alteration and two of target replacement were identified. More details are shown in the Supplementary Table S1.

In the case of virulence factors, *P. aeruginosa* AG1 has more than 250 genomic determinants for 11 classes or enriched groups, including adherence (flagella, type IV pili biosynthesis and motility), antimicrobial activity (phenazines biosynthesis), antiphagocytosis (alginate production), iron uptake (pyochelin and pyoverdine), enzymes (phospholipases), biosurfactant (rhamnolipid biosynthesis), quorum sensing, proteases, regulation of two component system, type three secretion systems (T3SS) and toxins (exotoxin-A). More details are shown in the Supplementary Table S2.

In the study of the mobilome, diversity of elements were identified. At the genomic islands level, a total of 57 laterally acquired regions (size >10 kb) were identified (light blue in Fig. 3a), which correspond to drastic changes in the average GC composition. Six prophages (including two intact) were identified. The two complete integrons already described were also found. In correspondence to this diversity of mobile elements, no complete/functional CRISPR-Cas systems were recognized.

Using BLASTn, RIVM-EMC2982 (Accession CP016955.1; 7,380,063 bp, 65.7% GC content and Prokka annotation: 6,871 CDS, 76 tRNAs, 1 tmRNA and 12 rRNA; ST-111 and blaVIM-2+) was identified as the closest genome to PaeAG1 (Query cover 99%, identity 100%), which is a ST-111 and blaVIM-2 carrying strain. Both strains have same number of RNAs genes. Synteny comparison of the nucleotide sequences of both strain revealed 99% identity and 92% of coverage comparing PaeAG1 strain against RIVM-EMC2982. In addition, comparison of genome of PaeAG1 (genome size of 7.2 Mb) was done against strains PAO1 (6.3 Mb) and RIVM-EMC2982 (7.4 Mb). As shown in Fig. 3c, genomic blocks contrast with the general reference of the *P. aeruginosa* group, PAO1, which has almost 1 MB of difference of the genome size and around 1 000 genes. In the case of comparison with RIVM-EMC2982, general profile by blocks found similar arrays between both strains, congruent with genome sizes and content of mobile determinants in both strains.

**Figure 4:**
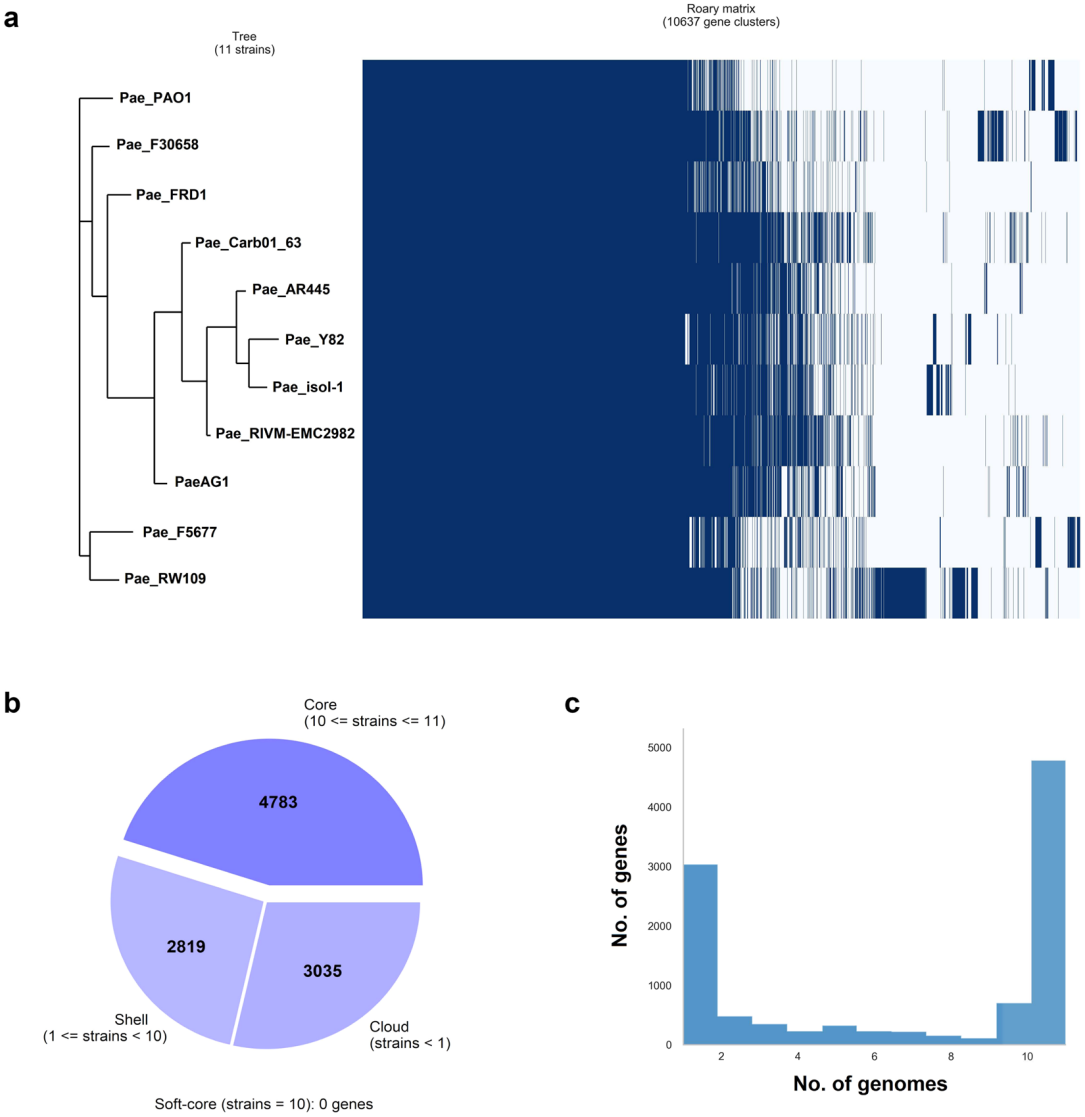
Pan-genome analysis of ST-111 *P. aeruginosa* strains. (**a**) Clustering according to strains profile by gene content. A total of 10,637 genes were identified. (**b**) Distribution of the gene content in all the strains, including that the core genome is composed of 4,783 (45% of total genes). Distribution of genes number by number of genomes is presented in (**c**).

In addition, comparison of gene content of ST-111 strains was used for phylogenetic analysis. A total of 9 complete genomes were available in PGDB, all with variable genome size (6.7–7.3 Mb) and gene content (6,200–7,400 genes). Pan-genome analysis revealed a total of 10,637 genes, which can separate strains in two clusters, one of them including PaeAG1 and *P. aeruginosa* RIVM-EMC2982 (Fig. 4a). The reference strain PAO1 was found to be completely separated from the group. Regarding core-genome, 4,783 genes (45% of total genes) were identified (present in at least 10 of the 11 sequences). A third part of genes were identified in only one of the strains. More details are shown in Fig. 4b,c. Interestingly, PaeAG1 is the only isolate which carries blaIMP-18 gene, in contrast to blaVIM-2 which was present in most of the strains.

## Discussion

*P. aeruginosa* is an opportunistic pathogen able to adapt to different environments and it causes a variety of acute and chronic infections. PaeAG1 is a clinical isolate from a Costa Rican hospital with a profile of multi-resistance to antibiotics. In this context, concern over the increasing prevalence in hospitals of high-risk clones, including *Pseudomonas aeruginosa*, has prompted the use of typing methods and sequencing strategies to study the genomic epidemiology of bacterial clones at high resolution^1^. Interested in the assembly and annotation of PaeAG1 genome, we implemented different approaches using short and long reads and we benchmark them using the 3C criterion.

### Benchmark of hybrid and non-hybrid assemblies

Of the more than 50 assemblies we run for pipeline standardization (considering different pre-processing, assembly and annotation steps), best cases per assembly were compared. In total 12 approaches were presented, and the best one was included as a 13^th^ case after polishing and curation. According to the global profiles given by metrics and 3C benchmark, variable results were obtained (Table 1 and Fig. 2).

Regarding contiguity, fewer contigs were assembled using long reads or hybrid approaches in comparison to short reads. As reported, assembly continuity and genome size seems not to be correlated^60^. This is verified in our case, and dependency on technology seems more evident. Also, dependency on algorithms showed different contiguity, even for same type of approach. Use of long reads (non-hybrid or hybrid method) improved contiguity metrics, solving most of conflictive regions that short reads could not assemble.

In the case of correctness, long reads only approaches presented critical problems in accuracy. As in our study, in a recent study error rates for short reads and hybrid assemblies were similar but were much higher for long reads assemblies using Unicycler in all cases^1^. Even though we had ultra-deep coverage for both sequencing technologies, this could be no enough to correct error in long reads only assemblies. This is probably due to systematic errors that have been detected in long reads sequencers, without compensation even increased sequencing depth^10^. In addition, our results using long reads only assemblers tended to have larger assemblies (total length) and duplication in different contigs was recognized. This is has been previously reported for long read assemblers^10^ and it could be a major obstacle for polishing the genome^12^ and compromising accuracy.

To assess completeness, we implemented an analysis using expected gene content by searching single-copy orthologs^61^. Short reads only and hybrid approaches achieved the assembly of 100% of core genes, but long reads only had a poor performance. Also, despite the larger number of CDS for long reads, incomplete assembly of genes was evidenced. Fragmentation of genes was confirmed by comparing the average size of all those elements. In long reads only assemblies the CDS average size was <600 bp, but for all other approaches this value was around 955 bp (Table 1). The CDS average size of the closest genome to PaeAG1, RIVM-EMC2982, is 955 bp, meanwhile for PAO1 strain is 1000 bp. This appreciation has been briefly reported before^62^. The incompleteness of genome assembly will not matter if genome structure is not the focus of a study^9^, but it is not the case of PaeAG1, where genomic events reconstruction would be crucial to understand the special features of this strain.

When all features of assemblies are included in the PCA analysis, general profiles of short reads approaches define a separated cluster, and another one for long reads and hybrid methods (Fig. 2a). Considering all the metrics of the 3C criterion, definitively SPAdes and Unicycler hybrid approaches outperformed non-hybrids methods. This can be explained due reference-free genomes assembly is feasible using best features of both short and long reads technologies^9^. IDBA assembler is a particular case which remains as the same using the hybrid or non-hybrid approach.

About other works related to the algorithms we evaluated, different results have been found depending on data and genome complexity. However, since introduction of Unicycler assembler, a last generation algorithm, most studies have suggested that Unicycler outperforms other approaches^1,10,11,63^. In the case of IDBA and Velvet, performance was comparable to SPAdes when it was introduced^36^. For Megahit, an assembler for metagenomes but also working for single genomes^38^, it has been also used in recent studies, mainly related to microbial communities or particular strains^64^. More restricted works using SKESA are reported, but performance seem to be better than SPAdes and Megahit for some cases^39^.

For short reads only or hybrid assemblies, SPAdes is still used to aseembly genomes^36,39,65^. In a recent study, SPAdes had better results when compared to others, where Unicycler was not included^3^.

For long reads, Canu has been successfully implemented in different studies^10,12,41^, showing well performance when benchmark is done (but most of them without Unicycler assember). For Flye, it has been used in recent studies^66,67^, including a case where Canu, Flye and Unicycler (using long reads only and hybrid approaches) had very similar performance^68^. Comparison between Unycicler, SPAdes and Canu has shown that in some cases Canu and SPAdes are not able to circularize the final assembly, unlike Unicycler^11^. In another study with long reads only, Canu was the best ranked assembler using *Escherichia coli* genome^12^.

All this variable results of assemblers (in our benchmark and the literature) are congruent with several reports about the diversity of assemblers, which have been developed to generate high quality *de novo* assemblies, but their output is very different because of algorithmic differences, data source and genomic complexity^2^. This complicates selection of appropriate strategy. Thus, the need for more capable assemblers is still mandatory in terms of capabilities, accuracy and the way to deal with genomic features^3^.

Regarding the differences in cost for both technologies (only considering sequencing step and no other complementary costs) Illumina short reads sequencing cost ($1500) was around three times more expensive than ONT ($500) sequencing. In our case, the hybrid approach has a cost of around $2000 for both technologies. Although we had ultra-deep sequencing data for both platforms, the minimal coverage requirements for PaeAG1 genome assembly are not known, which could significantly reduce the sequencing price. This cost is higher than other studies but with hundreds of sequenced samples^69,70^, in contrast with our case in that a single genome was sequenced (increasing costs).

In the case of conflictive regions, each assembler implements slightly different heuristics to deal with repetitions in the genome, uneven coverage, sequencing errors and chimeric reads^8^. Efforts to generate complete genome sequences with repetitive regions has been hampered by dramatic expansion of mobile elements, especially when short read sequencing methodologies are used^13^. In PaeAG1 genome assembly, different complicated regions were identified when short reads only approaches (all methods) and hybrid IDBA were used, creating gaps in an incomplete assembly (Fig. 2b). Although the PaeAG1 has not really a repeat-dense genome, mobile elements add repetitive sequences. This has complicated the assembly of its genome using short reads only approaches. All this regions were apparently solved by long reads only and for hybrid SPAdes and hybrid Unicycler. This results are expectable according to previous reports and the differences in each technology. Use of long reads technologies achieve repeat regions spanning^63^ and it permits bridging of repetitive sequences^65^.

However, evaluation of remapping of reads with the selected assembly (hybrid Unicycler according to 3C criterion) revealed a variation in the coverage in one specific region, as shown in Supplementary Fig. S1a (left), with an irregular and non-uniform distribution of reads. This conflictive region was preliminary annotated as a flanking repetitive sequence of one of the integrons (containing blaVIM-2 gene). This is a common phenomenon in regions carrying antimicrobial resistance determinants, which are often flanked by repetitive insertion sequences, and it can be difficult to assemble using short reads because are very short compared to the repetitions^10^. In our case, the conflictive region is part of the known region of the integron (approx. 2,500 bp, sequenced using Sanger method), and 100% of short reads had a size of 101 bp. Although this region was identified in a hybrid approach, this problem is an in force limitation of the algorithms^11^ and curation step was required.

No resolution of repetitive region made that short reads were mapped incorrectly^9^, evidenced as a coverage peak of reads in the remnant conflictive region of PaeAG1 genome assembly. In addition, this is congruent with the alignment of known sequence against the assembly. At least a 12% of the blaVIM-2 carrying integron sequence was lost in the hybrid approaches, including hybrid Unicycler (Table 1). We can conclude that those identical flanking regions of integrons were not well assembled using short reads. Long reads approaches were able to coverage both regions completely. The compromised ability of the Unicycler algorithm to assemble this conflictive region in the hybrid mode is related to the approach. In general, hybrid assembly can be accomplished with either a short-read-first or long-read-first approach. In the short-read-first method, contigs are assembled using short reads followed by a scaffolding is addresses using long reads^11^. Drawbacks of this approach include scaffolding mistakes and structural errors (misassemblies) in the sequence^71^. This could be the reason of our case in the conflictive region due Unicycler in hybrid mode is a short-read-first approach. In this context, the genome assembly problem is an open issue due is a NP-hard problem, and no universal solution to find the optimal route in graph-based approaches is available, in particular which is aggravated by repetitive regions. To deal with repetitive sequences in the genome, Unicycler determine the occurrence (multiplicity) of contigs in the assembly using both depth and connectivity using a greedy algorithm, and a bridging step is used to connect contigs and solve repeats using paired-end short reads^11^. However, due the algorithm used by Unicycler is a greedy approach, optimal solution is not warranted, and assembly errors can be induced. Thus, additional steps, as the manual curation, are required.

In this sense, manual curation is a common practice to finish genome due complexity of genomic data which algorithms not always can deal with^9,10^. In a case, by comparing long reads only and hybrid assemblies, this manual curation it implied recovery of lost sequences up to 18 kbp for some assemblies in another study^10^. Same situation was presented in another ST-111 *P. aeruginosa* strain, where flanking regions of blaVIM-2 gene was broken during assembly^72^. In other studies, no polishing strategy improves the completeness of assemblies^65^.

To improve the genome assembly of PaeAG1, curation was done with the help of the known sequences of PaeAG1 (Sanger sequencing), assembly graphs and the assemblies of long reads only. After this polishing step, remapping showed a uniform distribution of reads (Supplementary Fig. S1a right) and complete matching (100% identity and coverage) of the known sequences of the integrons, as expected.

At graph assembly level, when topological structure of assembly is analyzed for short reads assemblies (Fig. 2c, short reads), a collapsed graph is evidenced, where sequences are shown as cycles due the repeats or small shared sequences in many reads at same time. This means that there is insufficient information to disambiguate the repeat or shared sequences in the graph. This problem was solved when long reads were implemented, showing no cycles for long reads approaches (although shown case had two contigs), and a complete circularized genome for the final hybrid assembly.

### Assessment of the genome assembly of PaeAG1

Based on best overall quality statistics and polishing, hybrid approach using Unicycler was selected as the final assembly of PaeAG1 genome using 3C criterion.

In our initial efforts to assembly the genome, using only short reads, most of assemblers generated more than 100 contigs, and using RIVM-EMC2982 strain (which was selected after doing a full genome BLASTn of contigs), scaffolding finished with 1 sequence for the case of Unicycler and 22 gaps. In order to improve the genome assembly, ONT technology was used to produce long reads and new evaluations were made using both, long read only or hybrid methods.

On the other hand, notwithstanding all the three contiguity, completeness and correctness evaluation are frequently evaluated in genome assembly studies^3,8,12,15–17^, no explicit conceptualization of “3C criterion” has been achieved. Here we emphasized its use to referrer to the classical metrics and comparisons.

The final assessment of the definitive assembly of PaeAG1 genome accomplished an ultra-deep coverage for both, short (>400X) and long reads (>560X) technologies. Also it achieved high performance according to 3C criterion: (i) full contiguity with a single and circular genome without gaps; (ii) correctness based on short reads remapping and polishing, achieving full accuracy (including known sequences of the strain); and (iii) completeness according to identification of 100% of expected core gene set and percentage of remapping of genomic reads as well mapping of reads from RNASeq technology.

Altogether, the use of a hybrid strategy allowed the PaeAG1 genome to be inferred by a *de novo* or reference-free assembly approach, which it represent a key element in the study of this strain due its exclusive genomic features^9^. To our knowledge, this is the first genome assembly of a ST-111 *P. aeruginosa* strain using a hybrid approach.

The first hybrid assemblies for other-class *P. aeruginosa* strains were published recently^23,73,74^. In order to evaluate our pipeline in these publicly available sequencing data, we implemented our hybrid approach to the two cases with Illumina and ONT sequencing technologies. For the case of the *P. aeruginosa* strain Houston-1^73^, we were able to reproduce the assembly of the chromosome and the plasmid with our approach. For the *P. aeruginosa* strain CRPA^23^, the published draft genome was composed of three contigs, and with our approach we were able to finish into two contigs, representing an improvement in the assembly. More details of the assemblies of these two strains are shown at the end of the Supplementary Material Manual curation.

### Annotation of the PaeAG1 genome and epidemiological insights

In order to identify main features of the PaeAG1 genome, including its architecture, composition and functions, genome characterization and annotation was done. The PaeAG1 chromosome is a large and circular sequence of 7,190,208 bp, larger than reference strain PAO1 and similar to other ST-111 strains size^31,75^. Same pattern was found for the GC content of 65.7%. This relatively large genome in *P. aeruginosa* has been associated to thrive in a repertoire of hosts and environments^21^.

The general annotation of genome revealed that PaeAG1, contain 6,709 genes (including 6,620 CDS), which are related to 2,197 Gene ontology terms, 3,060 elements in KEGG and 5,537 COGs. In similar way as reported in first whole genome sequencing of a *P. aeruginosa* strain^76^, genome analysis of PaeAG1 shows determinants associated to versatility and successful ability to conquer multiple niches in nature. For example broad capabilities to transport and metabolize organic substances, presence of chemotaxis systems, biofilms production and efflux systems have been described and all of them were annotated for PaeAG1.

Genome sequence analysis using molecular typing methods showed that PaeAG1 has a ST-111 profile and O12 serotype. ST-111 is a lineage that belongs to the O12 serotype, which has been associated with multidrug resistance and expansion in hospitals for decades^28,72,75^. Thus, emergence of high-risk clones, including the ST-111 clones of *P. aeruginosa*, undermines the available therapeutic strategies and therefore, compromises public health. The presence of this kind of high-risk clones in Costa Rican hospitals is a nationwide concern because MBL and particular virulence factors producing isolates cause serious infections that are difficult to treat^77^. This same ST-111 profile has been identified in most of MBL producing *P. aeruginosa* strains in the United Kingdom^75^ thus as in Netherlands^77^.

Annotation of virulence factors found classical elements in *P. aeruginosa* group^78^, including elements related to adherence, antiphagocytosis, iron uptake, phospholipases, biosurfactant, quorum sensing, proteases, regulation, secretion systems, and toxins. Some particular virulence factors of PaeAG1 are substrate for type I protein secretion system T1SS (alkaline protease aprA), T2SS (elastases LasA and LasB, exotoxin-A and phospholipases PlcH, PlcN, and PlcB) and T3SS (ExoS, ExoT, and ExoY)^78^. It has been reported that secretion of ExoS is predominantly identified in invasive *P. aeruginosa* strains^78^. Recently, this determinant was identified in two blaVIM-2 carrying strains, one serotype O12 and ST-111 isolate (*P. aeruginosa* Carb01 63) and another O11 strain of ST-446 (*P. aeruginosa* S04 90) in Netherlands^31^. In PaeAG1, a potential invasive role of this strain can be related to the presence of this element.

In the context of mobile genetic elements, large number of determinants were identified in the chromosome of PaeAG1, including multiple genomic islands, six prophages and two integrons. Comparison of PaeAG1 against the reference of the *P. aeruginosa* group PAO1 and the closest strain to PaeAG1, RIVM-EMC2982, is consistent with genome size and mobile elements content. In the case of strain PAO1, this reference has a 6.3 Mb genome, meanwhile PaeAG1 has almost 1 Mb more of bases pairs (around 1,000 genes). This difference is congruent with high content of genomic island and other mobile elements in PaeAG1 but it is compromised in PAO1 strain. In the case of RIVM-EMC2982 (ST-111 and blaVIM-2+), this strain was identified as the closest to PaeAG1 and similar profile by genomic blocks were recognized (Fig. 3c). Meticulous analysis showed some different genomic arrangements, including differences in composition of mobile elements and absence of blaIMP-18 in RIVM-EMC2982.

In the case of the six prophages, all of them are also found in RIVM-EMC2982 genome (ten prophages in total) in same conditions of integrity. However, there are variable results of prophage presence in many ST-111 strains, which has been discussed as difficult to interpret, due transient nature of phages or the more methodological issues^72^. In addition, these high numbers of prophages might be related to the absence of CRISPR-Cas systems in the genome^31^, as the case of PaeAG1. Reports of compromised CRISPR-Cas defense systems are associated to better ability to acquire mobile element carrying antibiotic resistance genes in *P. aeruginosa* and other organisms^79^.

Regarding the integrons of PaeAG1, identification of genes *intl*1, *sul*1 and *qacEΔ*1 for class I integrons, suggested two integron-like structures carrying the VIM-2 and IMP-18 genes^22^. This was confirm when Sanger method was used for sequencing both integrons. In our assembly, these two complete integrons and same structure were found, one carrying blaVIM-2 and another one including blaIMP-18. This is congruent with previous studies showing that these two genes are regularly identified in integrons in *P. aeruginosa*^30,31,80^.

In more detail, VIM (Verona integron-encoded metallo-β-lactamase) enzymes have same hydrolytic spectrum than the IMP-type enzymes, and specifically blaVIM-2 is responsible of multiple outbreaks being the most widespread MBL in *P. aeruginosa*^30^. Multiple strains carrying VIM-2 have been identified in different latitudes around the world^75,80–83^. In United Kingdom, a study with 87 ST-111 *P. aeruginosa* strains found that 73 isolates carried VIM-2 and others carried different IMPs and one isolate had both VIM-2 and IMP-18, the second report of a clone carrying both MBL^75^. In a Netherlands outbreak, another strain (Carb01–63 strain, isolated from drains and sinks in a hospital) had a ST-111 profile and it was closely related to same RIVM-EMC2982^31^. All the three strains (PaeAG1, Carb01–63 and RIVM-EMC2982, in the same group according to phylogenetic analysis) are resistant to multiple antibiotics and carry blaVIM-2 allele.

In the case of imipenemases coded by blaIMP-18 gene, outbreaks reports and genetic context is limited in *P. aeruginosa*, including some cases in United States^84^, México^85^, France^81^ and Puerto Rico^86^.

For other antibiotic resistance determinants, annotation also included serine- and metallo-β-lactamases (PDC-3, OXA-2, as well as VIM-2 and IMP-18), porins and efflux pumps (including mexAB–oprM, mexCD–oprJ, mexEF–oprN, mexHI–opmD operons). All of them may contribute to the multi-resistance phenotype in PaeAG1.

As it was revealed by pan-genome analysis of ST-111 members, variable composition of gene content separate strains in relatively independent groups. The strains (including PaeAG1) belongs to the O12 serotype, which has been associated with multidrug resistance and nosocomial expansion^28,29^. PaeAG1 was close to the main group with 5 isolates, including the *P. aeruginosa* RIVM-EMC2982 (the closest to PaeAG1 by BLAST analysis) and Carb01–63 strains. Although all the strains (except the reference) are part of same group, differences in gene content is a remarkable feature, including that PaeAG1 was the only strain carrying blaIMP-18 genes. In contrast, ST-111 strains has been frequently associated with blaVIM-2, as mentioned before^28,75^. Other less commonly associated lactamases genes include VIM-4 or other IMP-type enzymes, but also only with extended-spectrum β-lactamases without carbapenemase activity (such as VEB-1 and OXA)^75^.

Due differences in size of the genome (6.7–7.3 Mb) and gene content, as well as the particular genomic features of this strains (genomic island composition and evolution, mobile elements, integrons, phages and others), further analysis are required to describe high plasticity in this group.

## Conclusions

Advances in sequencing technology play an increasing and determinant role in infection investigations and tracking evolution of international lineage of high-risk bacterial clones in clinical context over long times and in great detail^87^. However, genome assembly is not obvious and it is challenged by sequencing technology, genomic features and all bioinformatic algorithms, making it an open problem. Exhaustive comparison of different strategies to assembly the genome and it assessment gives a better way to get close to the real genome sequence. Benchmarking using the 3C criterion is a consensus approach that includes different levels and aims of comparison for the robust selection of a final assembly.

In our case, a hybrid assembly was the best approach to achieve a single circular sequence with high quality 3C for the case of the genome of a high-risk *P. aeruginosa* strain. Thus, best features of short and long reads sequencing technologies are included and their drawbacks are compensated.

The case of PaeAG1 genome assembly is a first and important step to understand the genomic architecture of an ST-111 high-risk strain. Annotation could reveal all the genomic content and molecular determinants related to phenotypes, which for PaeAG1 are related to multi-resistance and virulence mainly. This highlighting the need for more studies using epidemiological information and both high throughput technologies and conventional methods to understand the molecular mechanisms and phenotypes, make decisions at clinical level and to fight, and hopefully, overcome the antibiotic multi-resistance problem.

## Supplementary information

Supplementary information.

Supplementary information2.

Supplementary information3.

Supplementary information4.

Supplementary information5.

Supplementary information6.

Supplementary information7.

Supplementary information8.

## Data Availability

Data input and output data for PCA are provided as Supplementary material PCA data. The details of the approach for the manual curation are available in the Supplementary Material Manual Curation. Scripts for bioinformatics analysis are provided as a supplementary material, but also available at https://github.com/josemolina6/PaeAG1_genome/blob/master/Script_for_bioinformatic_analysis.sh. To specifically run the analysis of the 3C criterion, access a simplified Script at: https://github.com/josemolina6/PaeAG1_genome/blob/master/Script_3C_evaluation.sh. The annotated final assembly of the PaeAG1 chromosome was deposited in GenBank under the accession number CP045739. Short reads and long reads raw data were uploaded to the NCBI Sequence Read Archive (SRA) and it is available under the accessions numbers SRX7088413 and SRX7088414, respectively. A full table of all the details of the genome annotation is provided as a Supplementary material, and it is also available at: https://github.com/josemolina6/PaeAG1_genome. Files of the annotation in different formats as well as the fasta files of all the assemblies are available in the same link.
