# Supplementary material for: High quality 3C de novo assembly and annotation of a multidrug resistant ST-111 Pseudomonas aeruginosa genome: Benchmark of hybrid and non-hybrid assemblers
Source: Sci Rep. 2020 Jan 29;10:1392. doi: 10.1038/s41598-020-58319-6 (PMC6989561; doi:10.1038/s41598-020-58319-6)
Supplement: Supplementary file 1 — Supplementary information. [file 41598_2020_58319_MOESM1_ESM.docx]

Table S2. Detailed genomic determinants associated to virulence.

| **Class** | **Virulence**  **factors** | **Genes** |
| --- | --- | --- |
|  |  |  |
| Adherence | Flagella | flaG, fleN, fleQ, fleR, fleS, flgA, flgB, flgC, flgD, flgE, flgF, flgG, flgH, flgI, flgJ, flgK, flgL, flgM, flgN, flhA, flhB, flhF, fliA, fliC, fliD, fliE, fliF, fliG, fliH, fliI, fliJ, fliK, fliL,fliM, fliN, fliO, fliP, fliQ, fliR, fliS, fliT, motA, motB, motC, motD, motY |
|  | LPS O-antigen (*P. aeruginosa*) | - |
|  | Type IV pili biosynthesis | fimT, fimU, fimV, pilA, pilB, pilC, pilD, pilE, pilF, pilM, pilN, pilO, pilP, pilQ, pilR, pilS, pilT, pilU, pilV, pilW, pilX, pilY1, pilY2, pilZ |
|  | Type IV pili twitching motility related proteins | chpA, chpB, chpC, chpD, chpE, pilG, pilH, pilI, pilJ, pilK |
| Antimicrobial activity | Phenazines biosynthesis | phzA1, phzA2, phzB1, phzB2, phzC1, phzD1, phzE1, phzF1, phzG1, phzH,phzM, phzS. |
| Antiphagocytosis | Alginate biosynthesis | alg44, alg8, algA, algC, algD, algE, algF, algG, algI, algJ, algK, algL, algX |
|  | Alginate regulation | algP/algR3, algQ, algR, algU, algW, algZ, mucA, mucB, mucC, mucD, mucE, mucP |
| Biosurfactant | Rhamnolipid biosynthesis | rhlA, rhlB, rhlC |
| Enzyme | Hemolytic phospholipase C | plcH |
|  | Non-hemolytic phospholipase C | plcN |
|  | Phospholipase C | plcB |
| Iron uptake | Pyochelin receptor | fptA |
|  | Pyochelin | pchA, pchB, pchC, pchD, pchE, pchF, pchG, pchH, pchI, pchR |
|  | Pyoverdine receptors | fpvA |
|  | Pyoverdine | pvdA, pvdD, pvdE, pvdF, pvdG, pvdH, pvdI, pvdJ, pvdL, pvdM, pvdN, pvdO, pvdP, pvdQ, pvdS, pvdY |
| Protease | Alkaline protease | aprA |
|  | Elastase | lasA, lasB |
|  | Protease IV | prpL |
| Quorum sensing | Acylhomoserine lactone synthase | hdtS |
|  | N-(3-oxo-dodecanoyl)-L-homoserine lactone QS system | lasI, lasR |
|  | N-(butanoyl)-L-homoserine lactone QS system | rhlI, rhlR |
| Regulation | GacS/GacA two-component system | gacA, gacS |
| Secretion system | Hcp secretion island-1 encoded type VI secretion system (H-T6SS) | clpV1, fha1, hcp1, icmF1, ppkA, pppA, vgrG1 |
|  | *P. aeruginosa* TTSS translocated effectors | exoS, exoT, exoY |
|  | *P. aeruginosa* TTSS | exsA, exsB, exsC, exsD, exsE, pcr1, pcr2, pcr3, pcr4, pcrD, pcrG, pcrH, pcrR, pcrV, popB, popD, popN, pscB, pscC, pscD, pscE, pscF, pscG, pscH, pscI, pscJ, pscK, pscL, pscN, pscO, pscP, pscQ, pscR, pscS, pscT, pscU |
| Toxin | Exotoxin-A (ETA) | toxA |
|  | Hydrogen cyanide production | hcnA, hcnB, hcnC |
