# Supplementary material for: High quality 3C de novo assembly and annotation of a multidrug resistant ST-111 Pseudomonas aeruginosa genome: Benchmark of hybrid and non-hybrid assemblers
Source: Sci Rep. 2020 Jan 29;10:1392. doi: 10.1038/s41598-020-58319-6 (PMC6989561; doi:10.1038/s41598-020-58319-6)
Supplement: Supplementary file 6 — Supplementary information6. [file 41598_2020_58319_MOESM6_ESM.docx]

High quality 3C *de* *novo* assembly and annotation of a multidrug resistant ST-111 *Pseudomonas aeruginosa* genome: Benchmark of hybrid and non-hybrid assemblers

José Arturo Molina-Mora, César Rodríguez, Rebeca Campos-Sánchez, Leming Shi & Fernando García

Supplementary Material: Manual curation steps


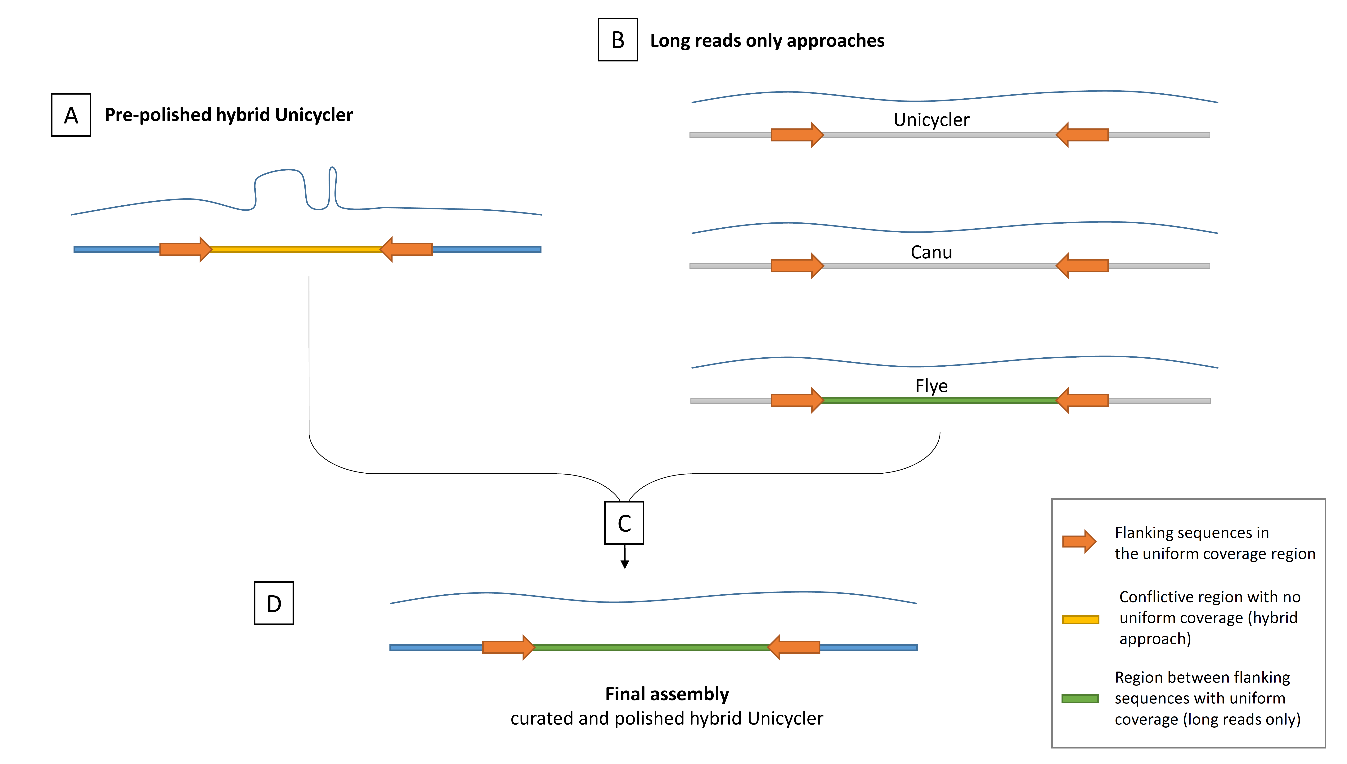


**Figure MC1.** Steps for the manual curation of the PaeAG1 genome assembly

**Steps:**

Figure MC1 shows the main four steps (A-B-C-D) related with the manual curation of the assembly.

1. Long reads were re-mapped to the Hybrid Unicycler assembly and a NOT-uniform coverage was found (step A) in the region 3,527,585-3,529,210 with a 2-fold coverage increase specifically in fragments 3,527,585-3,528,174 and 3,529,103-3,529,210 (Supplementary Fig. S1-A).
2. Flanking sequences were selected (arrows in Step A) in regions with uniform coverage and then they were identified in the other assemblies (long reads only approaches, arrows in step B).
3. A uniform coverage was found for the three long reads only assemblers (Canu, flye and Unicycler), without any conflictive region (at least in the middle of the Flanking sequences) as shown in step B. The three long reads only assembled obtained a similar sequence with less than 2% of bases were different (identity around 98%). Due Flye was the only one method with a single contig, it was used for the manual curation of the Hybrid Unicycler assembly.
4. The conflictive region in the hybrid Unicycler assembly was replaced by the equivalent sequence obtained by Flye (step C). The region included two identical segments which were not solved by Unicycler in the hybrid mode.

Sequence level analysis in the case of PaeAG1: As shown, the repeated sequence makes the hybrid approach to jump directly to the second gray sequence, meanwhile in the long read-only, this happens until for the second time the repeated sequence appears.

**Colors:** Flanking sequences Assembled sequences by both long reads only and hybrid approaches

Repeated sequence Lost sequence in the hybrid approach

**Long reads only (Flye)**

CGTGCCGTGATCGAAATCCAGATCCTTGACCCGCAGTTGCAAACCCTCACTGATCCGCATGCCCGTTCCATACAGAAGCTGGGCGAACAAACGATGCTCGCCTTCCAGAAAACCGAGGATGCGAACCACTTCATCCGGGGTCAGCACCACCGGCAAGCGCCGCGACGGCCGAGGTCTTCCGATCTCCTGAAGCCAGGGCAGATCCGTGCACAGCACCTTGCCGTAGAAGAACAGCAAGGCCGCCAATGCCTGACGATGCGTGGAGACCGAAACCTTGCGCTCGTTCGCCAGCCAGGACAGAAATGCCTCGACTTCGCTGCTGCCCAAGGTTGCCGGGTGACGCACACCGTGGAAACGGATGAAGGCACGAACCCAGTTGACATAAGCCTGTTCGGTTCGTAAACTGTAATGCAAGTAGCGTATGCGCTCACGCAACTGGTCCAGAACCTTGACCGAACGCAGCGGTGGTAACGGCGCAGTGGCGGTTTTCATGGCTTGTTATGACTGTTTTTTGTACAGTCTATGCCTCGGGCATCAAGCAGCAAGCGCGTTACGCCGTGGGTCGATGTTTGATGTTATGGAGCAGCAACGATGTTACGCAGCAGGGCAGTCGCCCTAAAACAAAGTTAGATGCACTAAGCACATAATTGCTCACAGCCAAACTATCAGGTCAAGTCTGCTTTTATTATTTTTAAGCGTGCATAATAAGCCCTACACAAATTGGGAGTTAGACGGCTTTGAGCGTTTCGATCTTACCTGTGAAAGAACAAGACGCTGCCGACTGGCTAGCGCTGCGGAATCTTCTTTGGCTCGCGGATGATCACGCCTCGGAGATTGAGCAGTACTTCTCTGGTGGACTTGAGGGGCTTGTAGAAGTGCTCATCGCCCGTGATGCTACCGGCGCGGCTGTTGGGCATGTCGAACTCTCGATAAGACATGACTTGGAAGAACTCAAGGAATCAAGACCGGCTACATCGAAGGCCTTTATGTGGCCCCAAGCCATCGATCAACAGACCTTGTGAGGCGTTTCTTGCGTGAGTCCGAGAAGTGGGCCCTAGAACAAGGGTGCAGCGCATTTGCCTCAGACAGAAGTGATCGGGTCATCACGCACCGCAAGTTCGCAGGCAGCGCCGTCTAACAACTCGTTCAAGCCGAACCCGCTTCGCTCCGGCAACGGCGTGGCAGGTTAAGCTTGCCACGCCGCCGCCTCCACTATGCGGGTCGGCTTAACTCAGGCGTTATGCCGCACTCACCCCCATGGAGTTTTGATGTTCAAACTTTTGAGTAAGTTATTGGTCTATTTGACCGCGTCTATCATGGCTATTGCGAGTCCGCTCGCTTTTTCCGTAGATTCTAGCGGTGAGTATCCGACAGTCAGCGAAATTCCGGTCGGGGAGGTCCGGCTTTACCAGATTGCCGATGGTGTTTGGTCGCATATCGCAACGCAGTCGTTTGATGGCGCAGTCTACCCGTCCAATGGTCTCATTGTCCGTGATGGTGATGAGTTCTTTTTTTGATTGATACAGCGTGGGGTGCGAAAAACACAGCGGCACTTCTCGCGGAGATTGAGAAGCAAAATGGACTTCCTGTAACGCGTGCAGTCTCCACGCACTTTCATGACGACCGCGTCGGCGGCGTTGATGTCCTTCGGGCGGCTGGGGTGGCAACGTACGCATCACCGTCGACACGCCGGCTAGCCGAGGTAGAGGGGAACGAGATTCCCACGCACTCTCTAGAAGGACTCTCATCGAGCGGGGACGCAGTGCGCTTCGGTCCAGTAGAACTCTTCTATCCTGGTGCTGCGCATTCGACCGACAACTTAGTTGTGTACGTCCCGTCTGCGAGTGTGCTCTATGGTGGTTGTGCGATTTATGAGTTGTCACGCACGTCTGCGGGGAACGTGGCCGATGCCGATCTGGCTGAATGGCCCACCTCCATTGAGCGGATTCAACAACACTACCCGGAAGCACAGTTCGTCATTCCGGGGCACGGCCTGCCGGGCGGTCTAGAACTTGCTCAAGCACACAACGAATGTTGTAAAAGCGCACACAAATCGCTCAGTCGTTGAGTAGCAGGCAGATGCGGCATAACATGAAGTTGCAGCCGACCATCACTCCGCTGCGCTCCGTTCTGGCGGCTGAACTTCGGCGTTAGATGCACTAAGCACATAATTGCTCACAGCCAAACTATCAGGTCAAGTCTGCTTTTATTATTTTTAAGCGTGCATAATAAGCCCTACACAAATTGGGAGTTAGACGGCTTTGAGCGTTTCGATCTTACCTGTGAAAGAACAAGACGCTGCCGACTGGCTAGCGCTGCGGAATCTTCTTTGGCTCGCGGATGATCACGCCTCGGAGATTGAGCAGTACTTCTCTGGTGGATTTGAGGAGCCTGCAGAAGTGCTCATCGCCCGTGATGCTACCGGCGCGGCTGTTGGGCATGTCGAACTCTCGATAAGACATGACTTGGAAGAACTCCAAGGAATCAAGACCGGCTACATCGAAGGCCTTTATGTGGCCCCAAGCCATCGATCAACAGACCTTGTGAGGCGTTTCTTGCGTGAGTCCGAGAAGTGGGCCCTAGAACAAGGGTGCAGCGCATTTGCCTCAGACAGAAGTGATCGGGTCATCACGCACCGCAAGTTCGCAGGCAGCGCCGTCTAACAACTCGTTCAAGCCGAACCCGCTTCGCTCCGGCAACGGCGTGGCAGGTTAAGCTTGCCACGCCGCCGCCTCCACTCTGCCGCTTAGCTTGGCGTTAGATGCACTAAGCACATAATTGCTCACAGCCAAACTATCAGGTCAAGTCTGCTTTTATTATTTTTAAGCGTGCATAATAAGCCCTACACAAATTGGGAGATATATCATGAAAGGCTGGCTTTTTTCTTGTTATCGCAATAGTTGGCGAAGTAATCGCAACATCCGCATTAAAATCTAGCGAGGGCTTTACTAAGCTTGCCCCTTCCGCCGTTGTCATAATCGGTTATGGCATCGCATTTTATTTTCTTTCTCTGGTTCTGAAATCCATCCCTGTCGGTGTTGCTTATGCAGTCTGGTCGGGACTCGGCGTCGTCATAATTACAGCCATTGCCTGGTTGCTTCATGGGCAAAAGCTTGATGCGTGGGGCTTTGTAGGTATGGGGCTCATAATTGCTGCCTTTTTGCTCGCCCGATCCCCATCGTGGAAGTCGCTGCGGAGGCCGACGCCATGGTGACGGTGTTCGGCATTCTGAATCTCACCGAGGACTCCTTCTTCGATGAGAGCCGGCGGCTAGACCCCGCCGGCGCTGTCACCGCGGCGATCGAAATGCTGCGAGTCGGATCAGACGTCGTGGATGTCGGACCGGCCGCCAGCCATCCGGACGCGAGGCCTGTATCGCCGGCCGATGAGATCAGACGTATTGCGCCGCTCTTAGACGCCCTGTCCGATCAGATGCACCGTGTTTCAATCGACAGCTTCCAACCGGAAACCCAGCGCTATGCGCTCAAGCGCGGCGTGGGCTACCTGAACGATATCCAAGGATTTCCTGACCCTGCGCTCTATCCCGATATTGCTGAGGCGGACTGCAGGCTGGTGGTTATGCACTCAGCGCAGCGGGATGGCATCGCCACCCGCACCGGTCACCTTCGACCCGAAGACGCGCTCGACGAGATTGTGCGGTTCTTCGAGGCGCGGGTTTCCGCCTTGCGACGGAGCGGGTCGCTGCCGACCGGCTCATCCTCGATCCGGGGATGGGATTTTTCTTGAGCCCCGCACCGGAAACATCGCTGCACGTGCTGTCGAACCTTCAAAAGCTGAAGTCGGCGTTGGGGCTTCCGCTATTGGTCTCGGTGTCGCGGAAATCCTTCTTGGGCGCCACCGTTGGCCTTCCTGTAAAGGATCTGGGTCCAGCGAGCCTTGCGGCGGAACTTCACGCGATCGGCAATGGCGCTGACTACGTCCGCACCCACGCGCCTGGAGATCTGCGAAGCGCAATCACCTTCTCGGAAACCCTCGCGAAATTTCGCAGTCGCGACGCCAGAGACCGAGGGTTAGATCATGCCTAGCATTCACCTTCCGGCCGCCCGCTAGCGGACCCTGGTCAGGTTCCGCGAAGGTGGGCGCAGACATGCTGGGCTCGTCAGGATCAAACTGCACTATGAGGCGGCGGTTCATACCGCGCCAGGGGAGCGAATGGACAGCGAGGAGCCTCCGAACGTTCGGGTCGCCTGCTCGGGTGATATCGACGAGGTTGTGCGGCTGATGCACGACGCTGCGGCGTGGATGTCCGCCAAGGGAACGCCCGCCTGGGACGTCGCGCGGATCGACCGGACATTCGCGGAGACCTTCGTCCTGAGATCCGAGCTCCTAGTCGCGAGTTGCAGCGACGGCATCGTCGGCTGTTGCACCTTGTCGGCCGAGGATCCCGAGTTCTGGCCCGACGCCCTCAAGGGGGAGGCCGCATATCTGCACAAGCTCGCGGTGCGACGGACACATGCGGGCCGGGGTGTCAGCTCCGCGCTGATCGAGGCTTGCCGCCATGCCGCGCGAACGCAGGGGTGCGCCAAGCTGCGGCTCGACTGCCACCCGAACCTGCGTGGCCTATACGAGCGGCTCGGATTCACCCACGTCGACACTTTCAATCCCGGCTGGGATCCAACCTTCATCGCAGAACGCCTAGAACTCGAAATCTAACGTCCGTTCGGGCATCGAGGTCCATGTCGGGGTGGGACGGGC

**Hybrid approach (hybrid Unicycler)**

CGTGCCGTGATCGAAATCCAGATCCTTGACCCGCAGTTGCAAACCCTCACTGATCCGCATGCCCGTTCCATACAGAAGCTGGGCGAACAAACGATGCTCGCCTTCCAGAAAACCGAGGATGCGAACCACTTCATCCGGGGTCAGCACCACCGGCAAGCGCCGCGACGGCCGAGGTCTTCCGATCTCCTGAAGCCAGGGCAGATCCGTGCACAGCACCTTGCCGTAGAAGAACAGCAAGGCCGCCAATGCCTGACGATGCGTGGAGACCGAAACCTTGCGCTCGTTCGCCAGCCAGGACAGAAATGCCTCGACTTCGCTGCTGCCCAAGGTTGCCGGGTGACGCACACCGTGGAAACGGATGAAGGCACGAACCCAGTGGACATAAGCCTGTTCGGTTCGTAAACTGTAATGCAAGTAGCGTATGCGCTCACGCAACTGGTCCAGAACCTTGACCGAACGCAGCGGTGGTAACGGCGCAGTGGCGGTTTTCATGGCTTGTTATGACTGTTTTTTTGTACAGTCTATGCCTCGGGCATCCAAGCAGCAAGCGCGTTACGCCGTGGGTCGATGTTTGATGTTATGGAGCAGCAACGATGTTACGCAGCAGGGCAGTCGCCCTAAAACAAAGTTAGATGCACTAAGCACATAATTGCTCACAGCCAAACTATCAGGTCAAGTCTGCTTTTATTATTTTTAAGCGTGCATAATAAGCCCTACACAAATTGGGAGTTAGACGGCTTTGAGCGTTTCGATCTTACCTGTGAAAGAACAAGACGCTGCCGACTGGCTAGCGCTGCGGAATCTTCTTTGGCTCGCGGATGATCACGCCTCGGAGATTGAGCAGTACTTCTCTGGTGGATTTGAGGAGCCTGCAGAAGTGCTCATCGCCCGTGATGCTACCGGCGCGGCTGTTGGGCATGTCGAACTCTCGATAAGACATGACTTGGAAGAACTCCAAGGAATCAAGACCGGCTACATCGAAGGCCTTTATGTGGCCCCAAGCCATCGATCAACAGACCTTGTGAGGCGTTTCTTGCGTGAGTCCGAGAAGTGGGCCCTAGAACAAGGGTGCAGCGCATTTGCCTCAGACAGAAGTGATCGGGTCATCACGCACCGCAAGTTCGCAGGCAGCGCCGTCTAACAACTCGTTCAAGCCGAACCCGCTTCGCTCCGGCAACGGCGTGGCAGGTTAAGCTTGCCACGCCGCCGCCTCCACTATGCGGGTCGGCTTAACTCAGGCGTTATGCCGCACTCACCCCCATGGAGTTTTGATGTTCAAACTTTTGAGTAAGTTATTGGTCTATTTGACCGCGTCTATCATGGCTATTGCGAGTCCGCTCGCTTTTTCCGTAGATTCTAGCGGTGAGTATCCGACAGTCAGCGAAATTCCGGTCGGGGAGGTCCGGCTTTACCAGATTGCCGATGGTGTTTGGTCGCATATCGCAACGCAGTCGTTTGATGGCGCAGTCTACCCGTCCAATGGTCTCATTGTCCGTGATGGTGATGAGTTGCTTTTGATTGATACAGCGTGGGGTGCGAAAAACACAGCGGCACTTCTCGCGGAGATTGAGAAGCAAATTGGACTTCCTGTAACGCGTGCAGTCTCCACGCACTTTCATGACGACCGCGTCGGCGGCGTTGATGTCCTTCGGGCGGCTGGGGTGGCAACGTACGCATCACCGTCGACACGCCGGCTAGCCGAGGTAGAGGGGAACGAGATTCCCACGCACTCTCTAGAAGGACTCTCATCGAGCGGGGACGCAGTGCGCTTCGGTCCAGTAGAACTCTTCTATCCTGGTGCTGCGCATTCGACCGACAACTTAGTTGTGTACGTCCCGTCTGCGAGTGTGCTCTATGGTGGTTGTGCGATTTATGAGTTGTCACGCACGTCTGCGGGGAACGTGGCCGATGCCGATCTGGCTGAATGGCCCACCTCCATTGAGCGGATTCAACAACACTACCCGGAAGCACAGTTCGTCATTCCGGGGCACGGCCTGCCGGGCGGTCTAGACTTGCTCAAGCACACAACGAATGTTGTAAAAGCGCACACAAATCGCTCAGTCGTTGAGTAGCAGGCAGATGCGGCATAACATGAAGTTGCAGCCGACCATCACTCCGCTGCGCTCCGTTCTGGCGGCTGAACTTCGGCGTTAGATGCACTAAGCACATAATTGCTCACAGCCAAACTATCAGGTCAAGTCTGCTTTTATTATTTTTAAGCGTGCATAATAAGCCCTACACAAATTGGGAGATATATCATGAAAGGCTGGCTTTTTCTTGTTATCGCAATAGTTGGCGAAGTAATCGCAACATCCGCATTAAAATCTAGCGAGGGCTTTACTAAGCTTGCCCCTTCCGCCGTTGTCATAATCGGTTATGGCATCGCATTTTATTTTCTTTCTCTGGTTCTGAAATCCATCCCTGTCGGTGTTGCTTATGCAGTCTGGTCGGGACTCGGCGTCGTCATAATTACAGCCATTGCCTGGTTGCTTCATGGGCAAAAGCTTGATGCGTGGGGCTTTGTAGGTATGGGGCTCATAATTGCTGCCTTTTTGCTCGCCCGATCCCCATCGTGGAAGTCGCTGCGGAGGCCGACGCCATGGTGACGGTGTTCGGCATTCTGAATCTCACCGAGGACTCCTTCTTCGATGAGAGCCGGCGGCTAGACCCCGCCGGCGCTGTCACCGCGGCGATCGAAATGCTGCGAGTCGGATCAGACGTCGTGGATGTCGGACCGGCCGCCAGCCATCCGGACGCGAGGCCTGTATCGCCGGCCGATGAGATCAGACGTATTGCGCCGCTCTTAGACGCCCTGTCCGATCAGATGCACCGTGTTTCAATCGACAGCTTCCAACCGGAAACCCAGCGCTATGCGCTCAAGCGCGGCGTGGGCTACCTGAACGATATCCAAGGATTTCCTGACCCTGCGCTCTATCCCGATATTGCTGAGGCGGACTGCAGGCTGGTGGTTATGCACTCAGCGCAGCGGGATGGCATCGCCACCCGCACCGGTCACCTTCGACCCGAAGACGCGCTCGACGAGATTGTGCGGTTCTTCGAGGCGCGGGTTTCCGCCTTGCGACGGAGCGGGGTCGCTGCCGACCGGCTCATCCTCGATCCGGGGATGGGATTTTTCTTGAGCCCCGCACCGGAAACATCGCTGCACGTGCTGTCGAACCTTCAAAAGCTGAAGTCGGCGTTGGGGCTTCCGCTATTGGTCTCGGTGTCGCGGAAATCCTTCTTGGGCGCCACCGTTGGCCTTCCTGTAAAGGATCTGGGTCCAGCGAGCCTTGCGGCGGAACTTCACGCGATCGGCAATGGCGCTGACTACGTCCGCACCCACGCGCCTGGAGATCTGCGAAGCGCAATCACCTTCTCGGAAACCCTCGCGAAATTTCGCAGTCGCGACGCCAGAGACCGAGGGTTAGATCATGCCTAGCATTCACCTTCCGGCCGCCCGCTAGCGGACCCTGGTCAGGTTCCGCGAAGGTGGGCGCAGACATGCTGGGCTCGTCAGGATCAAACTGCACTATGAGGCGGCGGTTCATACCGCGCCAGGGGAGCGAATGGACAGCGAGGAGCCTCCGAACGTTCGGGTCGCCTGCTCGGGTGATATCGACGAGGTTGTGCGGCTGATGCACGACGCTGCGGCGTGGATGTCCGCCAAGGGAACGCCCGCCTGGGACGTCGCGCGGATCGACCGGACATTCGCGGAGACCTTCGTCCTGAGATCCGAGCTCCTAGTCGCGAGTTGCAGCGACGGCATCGTCGGCTGTTGCACCTTGTCGGCCGAGGATCCCGAGTTCTGGCCCGACGCCCTCAAGGGGGAGGCCGCATATCTGCACAAGCTCGCGGTGCGACGGACACATGCGGGCCGGGGTGTCAGCTCCGCGCTGATCGAGGCTTGCCGCCATGCCGCGCGAACGCAGGGGTGCGCCAAGCTGCGGCTCGACTGCCACCCGAACCTGCGTGGCCTATACGAGCGGCTCGGATTCACCCACGTCGACACTTTCAATCCCGGCTGGGATCCAACCTTCATCGCAGAACGCCTAGAACTCGAAATCTAACGTCCGTTCGGGCATCGAGGTCCATGTCGGGGTGGGACGGGC

1. After edition, a polishing step was run using Pilon. To verify the effect of the modification, long reads were re-mapped, as shown in Figure S1 (before and after polishing). A uniform coverage was obtained (step D).
2. In addition, a 10 Kb region containing the “corrected conflictive region” was taken from the final assembly: positions 3 522 000 – 3 532 000. This sequence was selected using Artemis and sequence alignment was done comparing all long reads only assemblies, and the raw Hybrid Unicycler assembly without polishing. As expected, all the three long reads only had a high coverage percentage (>99% for all cases) without fragmentations, meanwhile the unpolished assembly was not able to find a single sequence to cover the 10Kb sequence, also as expected. See Table MC1 for details. Although long reads only were able to deal with this region, other issues related to gene fragmentation (for the three assemblers), error rate (all) and no single contig (except for Flye) make that hybrid approach was selected according to 3C criterion.

**Table MC1.** Alignment details of the fixed conflictive region against long reads only and pre-polished hybrid Unicycler approaches

| **Comparison** | | **Covered region of the 10 Kb sequence** | **Region in the genome** | **Coverage** | **Identity** |
| --- | --- | --- | --- | --- | --- |
| *Hybrid approach* | *Final assembly (polished hybrid Unicycler)* | 1 – 100001  (a single fragment, as expected due is the source sequence) | 3522000- 3532000 | 100% | 100% |
|  | *Unpolished Hybrid Unicycler* | Larger fragment 1: 1-7209  *By other fragments:*  Fragment 2:  7680-10001  Fragment 3: 7108-7701, and others in parts due repetitive regions. | 3522000-3529208,  3529069-3531393  3527585-3528178 | Larger fragment: 72%,  Overlapping fragments can cover 99% | 99% by fragments |
| *Long reads only* | *Flye* | A single fragment,  1-10001 | 5262345- 5272388,  47 gaps | 100% | 99% |
|  | *Canu* | A single fragment,  1-10001 | 350858- 360829,  29 gaps | 100% | 99% |
|  | *Unicycler* | A single fragment,  1-10001 | 4615674- 4605679, 11 gaps | 100% | 99% |

**Other structural variants:**

During the assembly evaluation, due we had paired-end reads, we proceeded to evaluate the reads distribution to identify other structural variants. We run the inGAP (Qi & Zhao, 2011) and Anise & Basil (Holtgrewe, Kuchenbecker, & Reinert, 2015) pipelines. Both methods detect breakpoints (including insertion breakpoints) and other structural variants such as duplications from aligned paired reads in BAM/SAM format.

Although the analysis Anise & Basil identified only two variants in the pre-polished hybrid Unicycler assembly, they were discarded out by the filtering step. Any of them were close to the conflictive region.

**Implementation with other Illumina and Nanopore sequencing data in *P. aeruginosa* strains.**

In order to evaluate our selected pipeline (hybrid Unicycler) with other data, publicly available sequencing data were searched. To our knowledge, only three studies have used short and long reads technologies to assemble *P. aeruginosa* genomes, but from non-ST111 strains (see table below). Two of them delivered complete assemblies and the third gave rise to an assembly composed of three contigs.

| **Study and publication date** | **Sequencing technology and assembly details** | **Strain and Multilocus sequence typing (MLST)** |
| --- | --- | --- |
| (Spinler, Raza, Runge, & Luna, 2019)  October 24, 2019 | Illumina and Nanopore technologies, by hybrid Unicycler and polishing using Pilon.  A chromosome and a plasmid were fully assembled. | Multidrug-Resistant *Pseudomonas aeruginosa* Endemic **Houston-1 Strain**.  MLST: Not reported |
| (Yu et al., 2019)  June, 2019. | Illumina and Nanopore technologies,  However only long reads data were used for the genome assembly using Canu, resulting in 3 contigs. | Carbapenem-resistant *Pseudomonas aeruginosa* isolate **CRPA strain**.  MLST: ST-730. |
| (Magalhães, Senn, & Blanc, 2019)  February 28, 2019 | Illumina and PacBio tecnhnologies. A single circular contig was produced for the three strains. Minimus was used for assembly. | *Pseudomonas aeruginosa* isolates H25883, H26023, and H26027.  MLST: H25883 (ST-1076), H26023 (ST-253), and H26027 (ST-17). |

We implemented our pipeline to assembly genomes of *P. aeruginosa* strains which were sequenced using Illumina and Nanopore technologies (Houston-1 and CRPA strains).

For Houston-1 strain, the published assembly (Spinler et al., 2019) was obtained using hybrid Unicycler, as in our approach. We were able to reproduce the results with our pipeline, including the assembly of the chromosome and one plasmid, as shown in the graph of the assembly (Visualized using Bandage). Metrics (calculated using Quast and using the published assembly as reference) and coverage (shown for long reads, with Qualimap) were congruent with expected results. The coverage obtained for the plasmid was 2-fold respect the chromosome.


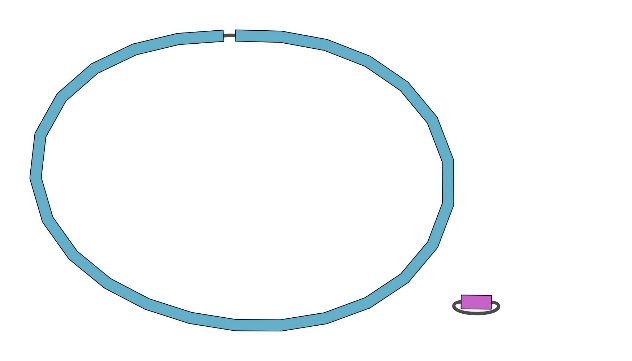

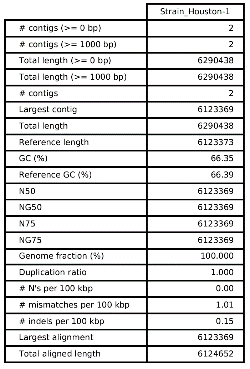

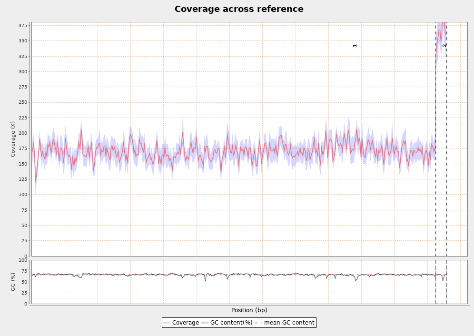


For the *P. aeruginosa* strain CRPA 23, the published draft genome was composed of three contigs (Yu et al., 2019). We run our pipeline and after contig assembly and scaffolding with the published assembly as reference, we were able to finish into two fragments (without Ns), representing a possible improvement in the assembly. Evaluation of reads re-mapping showed an uniform coverage (one extreme of one fragment had a 2-fold coverage, but also detected when the re-mapping was done using the published assembly). Further analysis are required to evaluate the impact of the change of contigs number on the annotation of this genome.


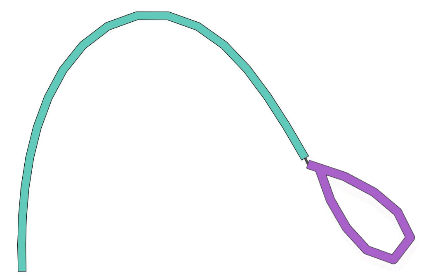

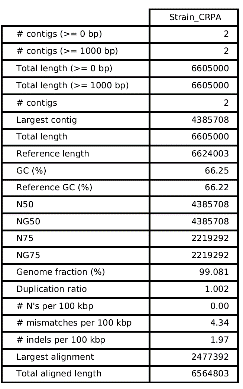

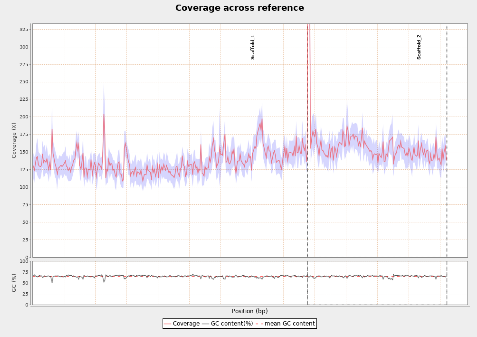


**References**

Holtgrewe, M., Kuchenbecker, L., & Reinert, K. (2015). Methods for the detection and assembly of novel sequence in high-throughput sequencing data. *Bioinformatics*, *31*(12), 1904–1912. https://doi.org/10.1093/bioinformatics/btv051

Magalhães, B., Senn, L., & Blanc, D. S. (2019). High-Quality Complete Genome Sequences of Three *Pseudomonas aeruginosa* Isolates Retrieved from Patients Hospitalized in Intensive Care Units. *Microbiology Resource Announcements*, *8*(9). https://doi.org/10.1128/MRA.01624-18

Qi, J., & Zhao, F. (2011). inGAP-sv: a novel scheme to identify and visualize structural variation from paired end mapping data. *Nucleic Acids Research*, *39*(suppl_2), W567–W575. https://doi.org/10.1093/nar/gkr506

Spinler, J. K., Raza, S., Runge, J. K., & Luna, R. A. (2019). Complete Genome Sequence of the Multidrug-Resistant Pseudomonas aeruginosa Endemic Houston-1 Strain, Isolated from a Pediatric Patient with Cystic Fibrosis and Assembled Using Oxford Nanopore and Illumina Sequencing. *Microbiology Resource Announcements*, *8*(43). https://doi.org/10.1128/MRA.00903-19

Yu, X., Han, Z., Ye, C., Zhou, S., Wu, S., Han, L., … Ye, H. (2019). Long-read Nanopore Sequencing-based Draft Genome of a Carbapenem-resistant Pseudomonas aeruginosa. *Journal of Global Antimicrobial Resistance*. https://doi.org/10.1016/j.jgar.2019.05.023
