# Supplementary material for: High quality 3C de novo assembly and annotation of a multidrug resistant ST-111 Pseudomonas aeruginosa genome: Benchmark of hybrid and non-hybrid assemblers
Source: Sci Rep. 2020 Jan 29;10:1392. doi: 10.1038/s41598-020-58319-6 (PMC6989561; doi:10.1038/s41598-020-58319-6)
Supplement: Supplementary file 8 — Supplementary information8. [file 41598_2020_58319_MOESM8_ESM.docx]

Table S1. Detailed genomic determinants associated to antibiotic resistance and phages.

| **Antibiotic resistance determinants** | | | |
| --- | --- | --- | --- |
| **Category by mechanism** | **Criteria** | **Hits** | **Genes** |
| Antibiotic target replacement | Perfect | 2 | sul1, sul1 |
|  | Strict | 0 | None |
| Antibiotic inactivation | Perfect | 4 | PDC-3, VIM-2, OXA-2, IMP-18 |
|  | Strict | 4 | APH(3')-IIb, catB7, fosA, OXA-50 |
| Antibiotic Efflux | Perfect | 14 | soxR, mexG, MexE, OprN, MuxC, MexA, mexH, emrE, MexF, CpxR, mexL, OprM, mexW, bcr-1.  oprD also presented but not identified by RGI (solved using BLASTp). |
|  | Strict | 30 | opmE, mexK, mexY, mexM, mexQ, TriA, mexI, MuxA, MexC, nalC, opmD, MuxB, mexJ, PmpM, TriC, mexR, MexD, MexT, mexN, MexB, mexP, OpmH, mexZ, Type A NfxB, OpmB, mexV, OprJ, nalD, MexS, TriB |
| Antibiotic target alteration | Perfect | 1 | soxR |
|  | Strict | 5 | mexR, gyrA, basR, basS, arnA |
| **Phages** | | | |
| **Completeness** | **Number** | **Total Proteins** | **Phage name** |
| Intact | 2 | 56 | PHAGE_Pseudo_JD024_NC_024330 |
|  |  | 46 | PHAGE_Pseudo_phi3_NC_030940 |
| Questionable | 2 | 26 | PHAGE_Pseudo_phiCTX_NC_003278 |
|  |  | 54 | PHAGE_Pseudo_F10_NC_007805 |
| Incomplete | 2 | 12 | PHAGE_Pseudo_PPpW_3_NC_023006 |
|  |  | 74 | PHAGE_Pseudo_JBD44_NC_030929 |
